# Disability health in medical education: development, implementation, and evaluation of a pilot curriculum at Stanford School of Medicine

**DOI:** 10.3389/fmed.2024.1355473

**Published:** 2024-09-04

**Authors:** Richard W. Sapp, Edmund Lee, Sylvia Bereknyei Merrell, Erika Schillinger, James N. Lau, Heidi M. Feldman, Cori McClure Poffenberger

**Affiliations:** ^1^Stanford University School of Medicine, Stanford, CA, United States; ^2^Department of Surgery, Stanford University School of Medicine, Stanford, CA, United States; ^3^Division of Primary Care and Population Health, Department of Medicine, Stanford University, Stanford, CA, United States; ^4^Division of Developmental-Behavioral Pediatrics, Department of Pediatrics, Stanford University School of Medicine, Stanford, CA, United States; ^5^Department of Emergency Medicine, Stanford University School of Medicine, Stanford, CA, United States

**Keywords:** disability, attitudes, medical students, education, medical education

## Abstract

**Background:**

People with disabilities face significant healthcare disparities due to barriers to accessing care, negative attitudes of providers, and lack of education on disabilities for healthcare professionals. Physicians report discomfort when interacting with patients with disabilities, adding to the disparity, warranting research on medical school education.

**Objective:**

Two educational interventions were structured: (1) a brief 2-h intervention in the mandatory curriculum and (2) a 9-week elective course which included interactions with individuals with disabilities through workshops and partner programs. We predicted that both of these interventions would result in improvements in attitude and empathy toward individuals with disabilities and reduce student anxiety.

**Methods:**

During the 2018–2019 academic year, 54 students completed the surveys for the 2-h intervention and 8 students completed the 2-h intervention and elective course. Pre-, post-, and delayed post-intervention surveys (3 months after post survey) measured students’ attitudes, using validated surveys on attitudes, empathy and anxiety toward individuals with disabilities.

**Results:**

Both educational interventions resulted in improved attitudes toward individuals with disabilities. However, students reported only feeling prepared to care for patients with disabilities after the elective course. The elective course, but not the 2-h course, significantly decreased student anxiety levels, likely due to more individual time working with individuals with disabilities. Delayed analysis after 3 months showed that both interventions had a lasting impact on attitudes and behavior change when caring for individuals with disabilities.

**Conclusion:**

Medical education is effective at improving medical students’ attitudes and behaviors toward individuals with disabilities. A 2-h session can lead to a modest improvement in attitudes. However, more dedicated time and exposure to persons with disabilities results in a greater improvement in students’ attitudes, anxiety and preparedness.

## Background

Disability affects 61 million people in the United States, or 1 in 4 (26%) people in the total population (1). This proportion is likely to increase as new clinical treatments and public health approaches prolong the lives of individuals with chronic conditions and disabilities. A significant proportion of individuals with disabilities require multi-specialty and complex care, and have been identified as frequent healthcare utilizers (2, 3). Physicians within all specialties care for patients with disabilities, and ideally can provide treatment with comfort and competence (4). However, current evidence suggests that there are significant healthcare disparities for individuals with disabilities, resulting from structural, socioeconomic, and attitudinal barriers that contribute to both inadequate access to care and poor quality of care (5, 6). Of these barriers, people with disabilities commonly report physician attitudes as a major obstacle when engaging with the US healthcare system (7–10).

Historically, medical education has paid limited attention to issues related to healthcare for individuals with disabilities (11–13). In the absence of explicit training, students may develop negative attitudes when working with individuals with disabilities (14). On the contrary, early and frequent encounters with individuals with disabilities may improve medical students’ knowledge, attitudes and skills regarding their care (15, 16). Despite a call to action for disability-based medical education from the US Institute of Medicine (17), the Office of the Surgeon General of the United States (18), and the Department of Health and Human Services (19), the lack of a curricular focus on disabilities remains the norm at many medical schools (11–13). Fortunately, curricula in disability have been developed in recent years, using a variety of methods including didactic lectures, home visits, and presentations in panels of individuals with disabilities. These methods have shown success in improving knowledge, skills and attitudes toward individuals with disabilities (20–27). Toolkits have been created to help integrate disability health into medical education and educational sessions have been created to address ableism and microaggressions (28–31). Within the past 4 years, more disability elective courses such as the one in our study have been developed and initiated by medical students (32). Additionally, disability competencies for healthcare education have recently been established by national consensus (33) and have been used to evaluate existing medical school curricula (34), finding a need for better integration of disability competency training throughout medical school education and training.

At Stanford School of Medicine in 2018–2019, we developed and implemented two novel disability-based medical education interventions to improve medical students’ knowledge, attitudes and skills pertaining to patient-centered care of people with disabilities: (1) a required 2-h session during the first-year mandatory curriculum and (2) a 9-week preclinical elective course with an incorporated patient partner program. The impact of these sessions on students’ knowledge, skills, and attitudes toward individuals with disabilities was measured through validated survey tools.

## Methods

### Educational interventions

Interventions were created with the motto “nothing about us without us in mind” (35, 36); individuals with disabilities were integrated and involved in every step of the development and implementation of the curriculum. Faculty, students, staff and individuals with disabilities worked together to create two-linked educational interventions: (1) Required 2-h session for first-year students as part of the first year Practice of Medicine (POM) course titled “Disability Health” and (2) 9-week elective course (Disability elective: Caring for Individuals with Disabilities).

### “Disability health” session

To develop this session, multiple meetings were held with faculty, students, and community members who identified with and without disabilities to determine the learning objectives and design of the session. The result of these discussions was a 3-part session: a brief lecture, panel discussion, and small group case discussions. The lecture, panel questions, and cases were created and reviewed by all members of the committee until consensus was met.

**Figure 1:**
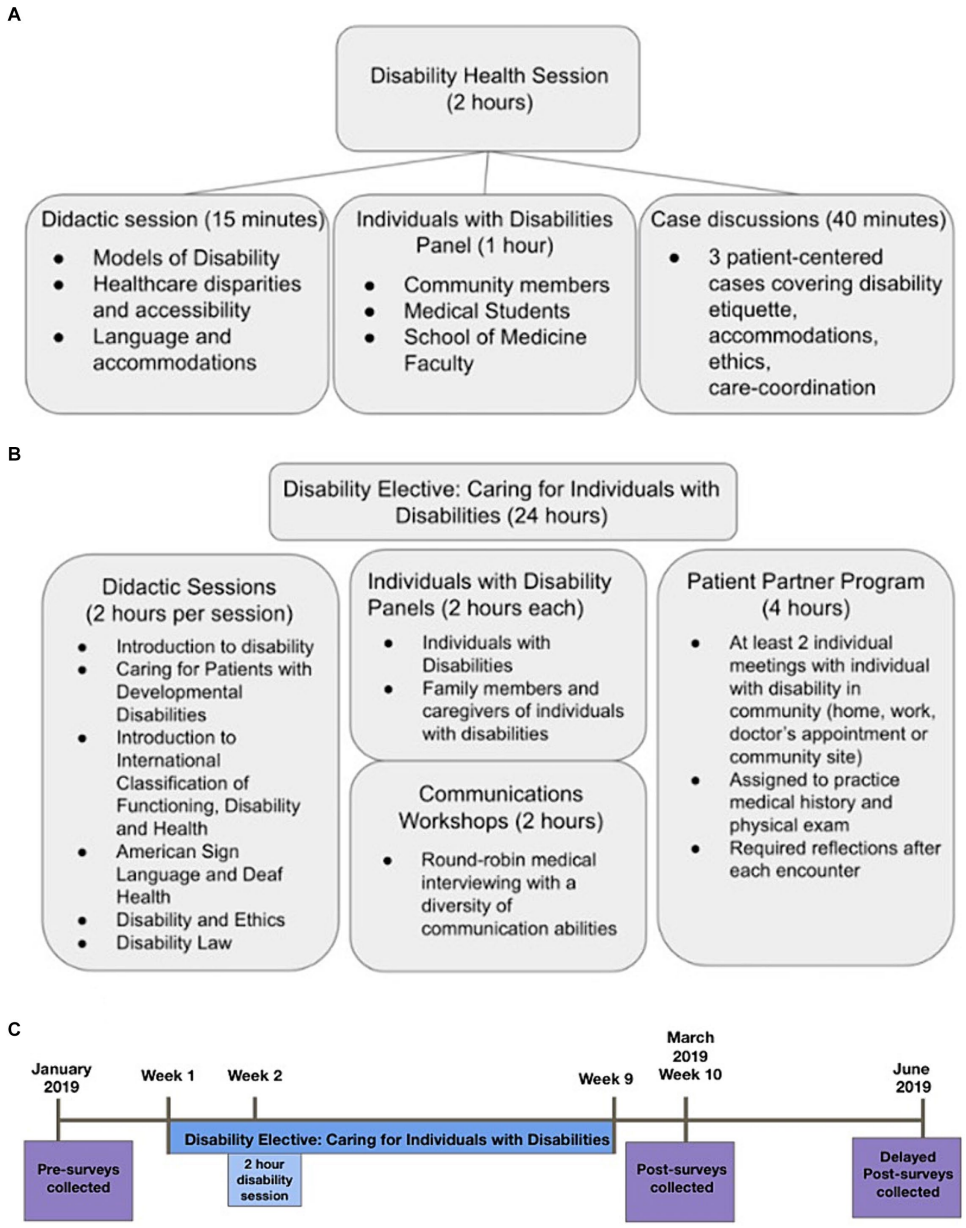
**(A)** Educational components of the 2 h disability health session. **(B)** Educational components of the disability elective course. **(C)** Timeline of survey administration.

The 2-h “Disability Health” session is a component of the first year Practice of Medicine (POM) curriculum, consisting of three parts: (1) a 15-min didactic session on healthcare disparities that individuals with disabilities face, the language around disability, and a comparison between the medical and social models of disability, (2) a 1-h panel with 5 local individuals with disabilities from both outside and within the school of medicine (faculty members, medical students and community members), representing different disabilities, ages, race/ethnicity, and functional strengths and needs, which focused on panelists’ positive and negative experiences within the healthcare system, along with advice for improving healthcare interactions, and (3) a 40-min case-based discussion involving three cases that discuss shared decision making, non-verbal communication, and disability etiquette (Figure 1A). Cases can be found in Supplemental Appendix 1. Case facilitators included the panelists, community members with disabilities, and medical school faculty with expertise in disability health. The overall learning objectives for the session were to: (1) define disability, (2) describe the relevance of the construct of disability to the practice of medicine, (3) contrast the medical model and social model of disability, (4) identify common challenges in providing health care for individuals with disabilities and discuss strategies for improvement, and (5) develop skills for inclusive conversations around disabilities.

### Disability elective

The 9-week elective course (24 h of content) (Figure 1B) was developed to expand on the major learning objectives from the Practice of Medicine session. The learning objectives were based on prior curricula (23) and from “Core Competencies on Disability for Health Care Education,” which was created by the Alliance for Disability in Health Care Education (33, 37). The overarching goals of the course were to: (1) to build general knowledge of common disabilities, and to dispel misconceptions and misunderstandings, (2) to instill attitudes and commitment to patient-centered care for people with disabilities, (3) to foster skills necessary for patient-centered care for people with disabilities. We worked with community members with disabilities to design the content of the curriculum based on the above goals. The schedule of the elective was the following: Week 1—Introduction to Disability, Week 2—Individuals with Disabilities Panel, Week 3—Caregivers of Individuals with Disabilities Panel, Week 4—Caring for a Patient with a Developmental Disability, Week 5—Communications Workshop, Week 6—Disability and Ethics, Week 7—International Classification and Functioning, Week 8—Disability Law, Week 9—ASL and Deaf Health.

The classroom components included seminar discussions, panel discussions with individuals with disabilities and parents/caregivers of individuals with disabilities, and a communication workshop (round-robin 15 min mini-medical history interviews with individuals with a diversity of communication abilities). Prior to Weeks 2 and 3, panelists reviewed and provided feedback on the structured discussion prompts. Participants in the communications workshop gave direct feedback to the medical students on their interactions during history-taking and physical exam practice. The community component included a patient-partner program in which students were paired with an individual with a disability in the community. Students were required to meet with their partner at least twice in two different locations (home, school, work, doctor’s office), and practice eliciting a history during one of those meetings. Individuals with disabilities provided insights about their disability experience, and students were required to write reflections after each meeting synthesizing their experiences.

### Participants

This study was conducted in a single private medical school in California (Stanford School of Medicine). Eligible individuals included preclerkship medical (MD) and physician assistant (PA) students (in their first or second year of professional school) who were 18 years or older. The study was approved by the Institutional Review Board (IRB) of Stanford University (IRB-47216).

### Survey content and measurement instruments

#### Demographics

Gender, age, ethnicity and training year were gathered at the beginning of data collection prior to the first survey. Demographic questions were taken from prior studies (38, 39). In addition, students were asked about their experiences with individuals with disabilities, including whether they self-identified as having a disability, a set of questions that had not been asked in the prior surveys.

The assessment was designed to assess the degree of change in attitudes, empathy, anxiety and competency. The survey was pretested and edited at the Goodman Surgical Education Center with experts in medical education and with individuals with disabilities who assisted in the development of the curriculum.

#### Attitudes

We used 2 existing questionnaires to assess attitude change as a function of education:

1. Medical Student Attitudes Toward Persons with Disabilities (MSATPD): is a 30-item questionnaire to measure medical students’ attitudes toward people with disabilities (38). It comprises six factual/demographic items and 24 opinion questions. It utilizes a 4-point Likert scale ranging from 1 (Strongly Disagree) to 4 (Strongly Agree), with a higher score indicating a more positive attitude. The Total Score was the sum of scores on individual items out of 92. Item 16 (“If I introduced a person with disabilities to my friends, I think they would feel uneasy”) was not analyzed due to it not loading onto any component during the psychometric content analysis, and there being a lack of pattern in participant responses (38). This instrument has been used to measure changes in medical students’ attitudes in two prior studies (15, 40). The scale demonstrates good internal consistency in this study (ɑ = 0.815).
2. Disabilities Attitudes in Health Care (DAHC) contains 17 items that include positively and negatively worded statements that address general attitudes, cost-effectiveness, time and energy, therapeutic potential, and educational preparation of healthcare professionals caring for people with disabilities (39). It utilizes a 5-point Likert scale ranging from 1 (Strongly Disagree) to 5 (Strongly Agree), with a higher score indicating a more positive attitude. Total score out of 85. This scale has been used to measure changes in medical students’ attitudes after an educational intervention (24). The scale demonstrates good internal consistency in this study (ɑ = 0.792).

##### Empathy

We relied on the Jefferson Scale of Physician Empathy Medical Student Version (JSE-S) to measure empathy among students of health professions. Participants are asked to indicate the extent of their agreement or disagreement with 20 different statements, ranging from 1 (Strongly Disagree) to 7 (Strongly Agree) (41). A higher number on the scale indicates higher agreement, which indicates higher levels of empathy (although it is noted that the JSE-S is not disability-specific). Total score out of 140. The scale demonstrates good internal consistency in this study (ɑ = 0.871).

##### Anxiety

This 12-item scale is a modified version of the intergroup anxiety scale (42) which was adapted toward individuals with disabilities (40). The response format employs a 10-point scale ranging from “Not at all” to “Extremely” on the following items: uncertain, worried, awkward, anxious, threatened, nervous, comfortable, trusting, friendly, confident, safe and at ease (the latter six are reverse scored). A lower score on this scale indicates lower levels of anxiety. Total score out of 120. The scale demonstrates good internal consistency in this study (ɑ = 0.896).

##### Additional questions

We added seven questions, adapted from a Survey Scale section “Interacting with the Disabled” and one question from the “Advocacy” scale (43). These questions were included to measure the self-reported level of competency and knowledge about interacting and working with individuals with disabilities. A group of faculty and students, experienced in medical education and survey design, modified existing questions and developed seven new questions regarding levels of confidence. Participants are asked to indicate the extent of their agreement from 1 (Strongly Disagree) to 5 (Strongly Agree). The higher score indicates higher agreement, indicative of greater competency and confidence in interacting with individuals with disabilities.

### Survey administration

Our study was completed via a survey hosted by Qualtrics^®^ (Provo, Utah, United States). We sent an email to participants with a link to the surveys, which led directly to an information page with an online consent form, followed by the measurement instruments stated above. Students developed their own personal identification codes to facilitate the pairing of responses while maintaining anonymity.

For the mandatory disability session, all first year MD and PA students were eligible for survey participation and were emailed the pre-survey in January 2019. The mandatory session took place 1 week after the pre-survey. Students received the post-survey 10 weeks after the mandatory session. The delayed post-survey was sent 20 weeks after the mandatory session (Figure 1C).

For the 9 week disability elective course, first and second year MD and PA students were eligible to participate. The same pre-survey was also sent in January 2019. First year MD and PA students enrolled in the disability elective took both the disability session and elective concurrently, and thus only filled out one survey (personal identification codes associated with the survey prevented duplicate responses). Students received the post-survey 1 week after the end of the disability elective course. The delayed post-survey was sent 12 weeks after the end of the elective course (Figure 1C).

### Data analysis

Survey responses were aggregated into Microsoft^®^ Excel (Redmond, Washington, United States) and analyzed using IBM SPSS Statistics Version 25 (Armonk, New York, US). We compared the baseline survey with the immediate and delayed post-education surveys for two groups of students: (1) those who took the 2-h required session in the Practice of Medicine Course only and (2) those who took the “Disability Health Session” and Disability elective. For analyzing the demographics, we used Chi-Square to determine differences between the groups that just took the mandatory 2-h disability session and those who took the disability session and disability elective. When analyzing pre-post intervention, and post, delayed post intervention, we used paired-T-test for the overall scores for the different measurement tools. On individual items on the surveys, we compared them using paired *t*-tests.

## Results

### Demographics

The total number of participants who attended the 2-h “Disability Health” session and completed all surveys was 54 (47% response rate). The total number of participants who attended the Disability elective was 8 (100% response rate). Demographic data of participants can be found in Table 1. There were noted differences in demographic data: (1) students who only took the disability health session had more professional work experience with people with disabilities (*p* = 0.045) and (2) Students who took both the disability health session and elective had more of a career interest in working with individuals with disabilities (*p* = 0.034).

**Table 1:** MD/PA student respondent demographic data.

	Disability health session	Disability elective
**Total n (%)**	54	8
*MS1*	38 (70)	3 (37.5)
*MS2*	0	5 (62.5)
*PA1*	15 (28)	0 (0)
*No response*	1 (2)	0 (0)
Sex		
*Male*	19 (35)	2 (25)
*Female*	33 (61)	6 (75)
*Other*	0 (0)	0 (0)
*Did not disclose*	2 (4)	0 (0)
**Mean age (years)**	24.9	26
Ethnicity		
*White*	26 (48)	4 (50)
*Black/African American*	2 (4)	0 (0)
*Asian*	20 (37)	4 (50)
*Native Hawaiian/Pacific Islander*	1 (2)	0 (0)
*Hispanic/Latino*	7 (13)	0 (0)
*American Indian/Alaska Native*	0 (0)	0 (0)
*Middle Eastern/North African*	2 (4)	0 (0)
*Another Race/Ethnicity/Origin*	0 (0)	0 (0)
*Did not disclose*	2 (4)	0 (0)
Professional/work experience caring for an individual with a disability		
*Yes*	19 (35)	0 (0)
*No*	35 (65)	8 (100)
Structure experiences working with people with disabilities (i.e., volunteering, teaching) aside from medical school?		
*Yes*	27 (50)	2 (25)
*No*	27 (50)	6 (75)
Friend or relative with a disability who you see at least occasionally		
*Yes*	34 (63)	5 (62.5)
*No*	20 (27)	3 (37.5)
Identify as having a disability		
*Yes*	7 (13)	2 (25)
*No*	47 (87)	6 (75)
Career interest in working with individuals with disabilities		
*None*	9 (16)	0 (0)
*Slight*	15 (28)	1 (12.5)
*Moderate*	21 (39)	3 (37.5)
*Strong*	8 (15)	4 (50)
*Very strong*	1 (2)	0 (0)
Level of training received in school regarding individuals with disabilities		
*None*	30 (56)	1 (12.5)
*A little*	20 (37)	7 (87.5)
*Somewhat*	4 (7)	0 (0)
*A lot*	0 (0)	0 (0)
*A great deal*	0 (0)	0 (0)

There were no reported differences in the pre-survey scores for any of the scales when taking into account of the survey respondents’ demographics: class year, sex, ethnicity, age, professional/work experience, volunteering, close relative or friend with a disability, self-identification with a disability, career interest or level of perceived training (data not shown).

### Disability health 2-h session

Students valued the session highly (4.1/5) and favored the patient panel (4.6/5) over the didactics (3.8/5) and cases (3.5/5). The class was successful at meeting the educational objectives for the students: (1) Define disability (3.9/5), (2) Describe the relevance of the construct of disability to the practice of medicine (4.1/5), (3) Contrast the medical model and social model of disability (4.1/5), (4) Identify common challenges in providing health care for individuals with disabilities and discuss strategies for improvement (4.2/5), and (5) Develop skills for inclusive conversations around disabilities (3.8/5). Students stated they felt they gained more awareness and a better understanding about how to talk about disability. 80% (42/54) of students were interested in further education on disability in the curriculum.

**Figure 2:**
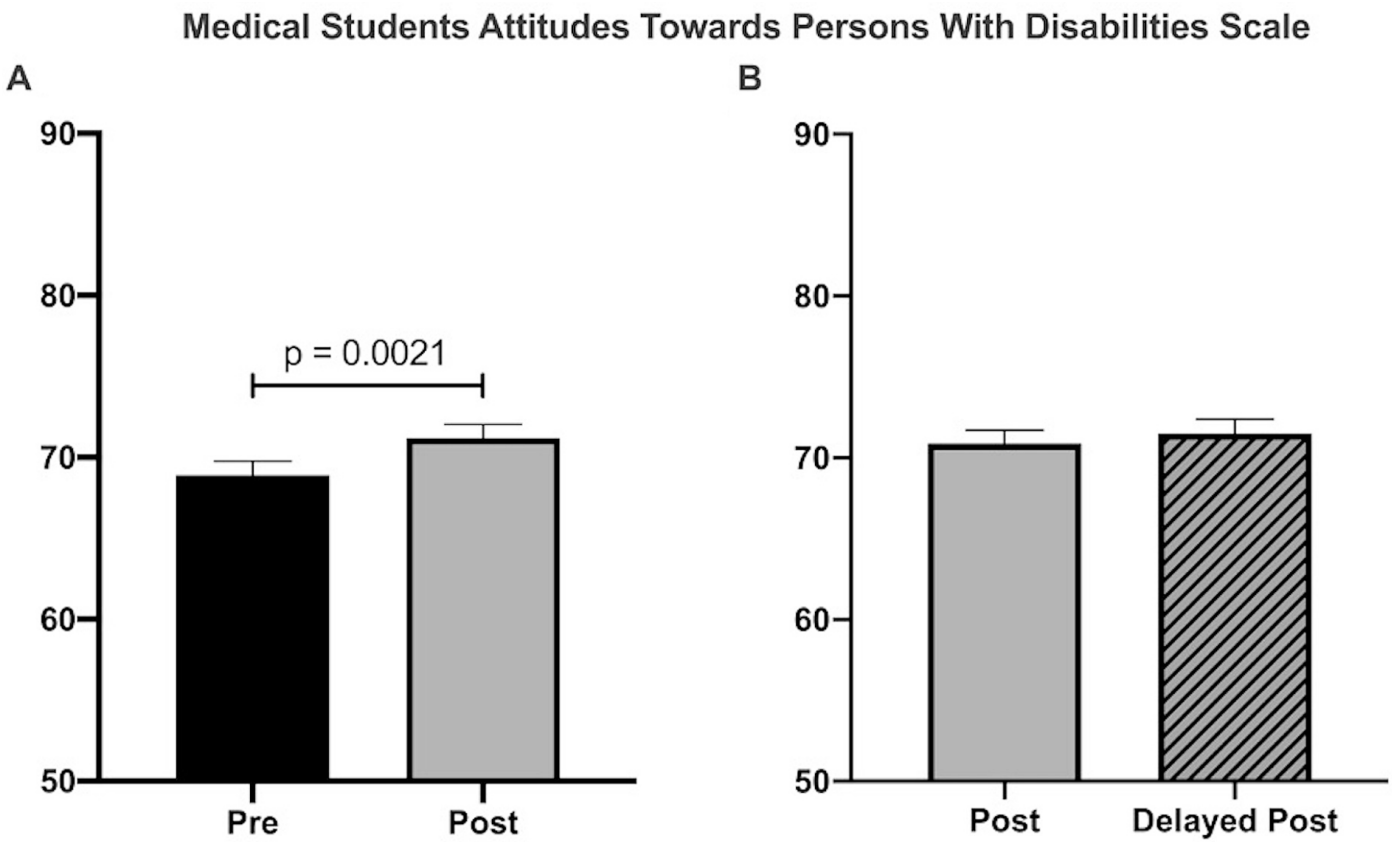
“Disability Health” 2-h session measured by the Medical Students Attitudes Toward Persons With Disabilities Scale. **(A)** There was a significant increase in attitudes toward persons with disabilities (68.9 vs. 71.2); *n* = 54, *p* = 0.0021, Students paired *t*-test. **(B)** There was no significant difference between post and delayed posttests (70.9 vs. 71.5, *n* = 49, *p* = 0.42, Students paired *t*-test).

Comparing the total scores on the Pre-vs. Post-scales for the students who received the required 2-h curricular intervention demonstrated a statistically significant increase on the “Medical Student Attitudes Toward Persons with Disabilities” scale (68.91 vs. 71.19, *p* = 0.0021) (Figure 2A) but no change on the Disability Attitudes in Health Care scale (DAHC), Jefferson Scale of Empathy (JSE) or Anxiety Scale (Supplementary Figures S1A–C; Supplementary Tables S2–S4). A delayed post-test was given 3 months after the administration of the initial post-test and there was no significant change on any of the scales (Figure 2B; Supplementary Figures S1D–F; Supplementary Tables S2–S4).

When looking at the specific items of the Medical Students Attitudes Toward Persons With Disabilities Scale, there were three statements which were significantly different indicating a more positive attitude toward individuals with disabilities: “Most people with disabilities feel sorry for themselves (reversed)” (3.13 vs. 3.32, *p* = 0.048), “People with disabilities are as happy as people without disabilities” (2.96 vs. 3.2, *p* = 0.05), and “Most people with disabilities resent people without disabilities (reversed)” (3.24 vs. 3.52, *p* < 0.0001). In Part B of the scenario section of the survey where they answer questions regarding a hypothetical scenario with a man with a disability and a woman of the same age in the room to be evaluate by a healthcare provider, the following statement was significant for students “In scenario B, I would be comfortable determining the role of the man vs. the woman in providing the history of the complaint” (Supplementary Table S1).

On the individual statements of the Disabilities Attitudes in Health Care survey, Jefferson Scale of Empathy, and Anxiety scale there were no significant differences comparing the pre-and post-test surveys (Supplementary Tables S2–S4).

Students were found to have significant improvements to their confidence in their understanding of “disability” and the barriers to healthcare access, barriers to participation and quality of life issues. The following statements were statistically significant: “I am comfortable providing assistance appropriately to a person with a disability” (3.39 vs. 4.17, *p* = 0.049), “I feel confident in my understanding of ‘disability’” (3.12 vs. 3.91, *p* < 0.0001), “I feel confident in knowledge of barriers to access to care for persons with disabilities” (2.7 vs. 3.56, *p* < 0.0001), “I feel confident in my understanding of cultural, economic, and physical barriers to participation” (2.7 vs. 3.4, *p* < 0.0001), and “I feel confident in knowledge about my understanding about the quality of life issues for people with disabilities” (2.6 vs. 3.41, *p* < 0.0001). It was found that there was a significant increase in the statement “I feel prepared to take care of patients with disabilities (2.07 vs. 2.48, *p* = 0.014), however, when looking at the absolute score students were reporting between neutral and disagree on this statement (2.48/5) (Supplementary Table S5).

### Disability elective

Overall, the students highly rated the overall value of the disability elective (4.75/5). They rated sessions with interactions with individuals with disabilities highly: Individuals with disabilities panel (4.625/5), Caregivers of individuals with disabilities panel (4.5/5), communications workshop (4.5/5) and partner program (4.75/5). Students spent an average of 4.625 h with their partner outside of class. Didactic sessions were also rated highly: Introduction to disability (4.167/5), Caring for a patient with a developmental disability (4.167/5), Disability and Ethics 4.0/5, International classification and functioning (3.625/5), Disability law/cases (5/5), and ASL/Deaf health (4.5/5).

**Figure 3:**
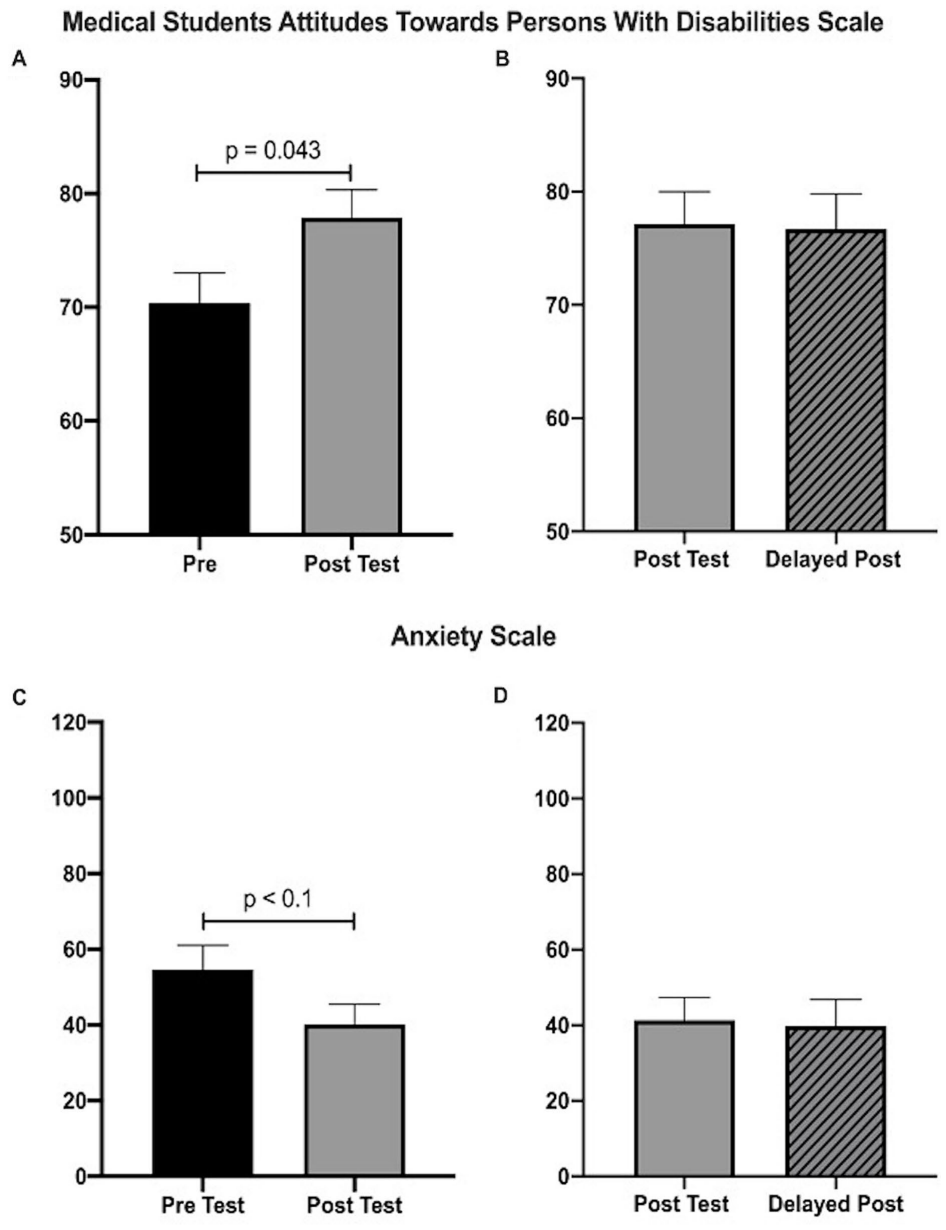
Disability Elective measured by Medical Students Attitudes Toward Persons with Disabilities Scale and Anxiety scale. **(A)** There was a significant increase in attitudes toward persons with disabilities as measured by the Medical Student Attitudes Toward Persons with Disabilities scale (70.4 vs. 77.8; *n* = 8, *p* < 0.05; Students paired *t*-test). **(B)** There was a marginally significant decrease in anxiety toward interacting with individuals with disabilities as measured by the Anxiety Scale (54.63 vs. 40.13; *n* = 8, *p* < 0.10; Students paired *t*-test). **(C,D)** There was no significant difference in post and delay post for either the Medical Students Attitudes Toward Persons with Disabilities Scale (77.14 vs. 76.85, *n* = 7, *p* = 0.76; Students paired *t*-test) and Anxiety scale (40.23 vs. 39.9, *n* = 7, *p* = 0.53; Students paired *t*-test).

Students who were enrolled in the 9 week disability elective demonstrated significant improvement in attitudes and decreased anxiety when comparing pre and post-tests. On the Medical Students Attitudes Toward Persons with Disabilities scale, there was a significant improvement in attitudes (70.4 vs. 77.8; *p* = 0.043) (Figure 3A) but not on the DAHC scale (Supplementary Figure S2A). Students had significantly decreased anxiety toward individuals with disabilities based on the anxiety scale (54.63 vs. 40.13; *p* < 0.1). There was no difference in the total score of the Jefferson Scale of Empathy (Supplementary Figure S2B). Three months after the post test, a delayed posttest was given and there was no significant change on any of the scales (Figures 2D, 3B; Supplementary Figures S2C,D).

On the Medical Students Attitudes Toward Persons with Disabilities scale, there were two significant statements demonstrating a more positive attitude toward individuals with disabilities “I would be comfortable interacting with a person with an intellectual disability who was in the community on his or her own (i.e., without staff members or caretakers)” (2.875 vs. 3.625, *p* = 0.048) as well as “In Scenario B, I would be comfortable determining the role of the man vs. the woman in providing the history of the complaint” where the man has a disability (2 vs. 3.625, *p* < 0.018) (Supplementary Table S6).

There were no individual items of significance on the Jefferson Scale of Empathy (Supplementary Table S8) and specific items on the Anxiety Scale trended toward significance, which included being less “nervous,” “uncertain” and “worried,” and being more “friendly” (Supplementary Table S9).

Students in the elective course had significant improvement on the comfort and confidence on individual items when comparing the pre and post surveys (Supplementary Table S10). In regards to the statement “I feel prepared to take care of patients with disabilities,” students felt more prepared after the disability elective (2 vs. 3.625, *p* = 0.006, Supplementary Table S10). In addition, students scored significantly higher on their understanding of patients with disabilities and their knowledge on barriers to access of care: “I feel confident in my understanding of ‘disability’” (3.25 vs. 4.13, *p* = 0.04), “I feel confident in knowledge of barriers to access to care for persons with disabilities” (2.38 vs. 4.38, *p* = 0.01), and “I feel confident in my understanding of cultural, economic, and physical barriers to participation” (2.88 vs. 4.50, *p* = 0.02). On survey items related to comfort in performing aspects of a history and physical exam with a patient with a disability, students performed significantly higher on the following statements: “I am comfortable adapting my body positions to make someone who uses a wheelchair more comfortable” (2.88 vs. 4.25, *p* = 0.01), “I am comfortable adapting my body positions to facilitate effective communication for someone who is visually or hearing impaired” (3.63 vs. 4.75, *p* = 0.03), “I am comfortable adapting my interviewing technique to accommodate patients with disabilities” (2.75 vs. 4.25; *p* = 0.01) and “I feel confident in communicating with patients with disabilities” (2.63 vs. 4.25, *p* = 0.02).

### Comparison of educational interventions (disability health session vs. disability elective)

In comparing the baseline pre-survey scores between both educational interventions, there was no statistical difference on any of the survey instruments (Table 2).

**Table 2:** Data table summarizing the measurement instruments across the educational interventions.

	Pre-survey	Post-survey	Pre- vs. Post	Delayed post-survey	Post vs. Delayed post
Medical student attitudes toward persons with disabilities (MSATPD)					
Disability session (*n* = 54)	68.9	71.2	***p* = 0.0021**	71.5	*p* = 0.42
Elective course (*n* = 8)	70.4	77.8	***p* = 0.043**	76.85	*p* = 0.76
Session vs. Elective	*p* = 0.28	***p* = 0.006**		***p* = 0.05**	
Disabilities attitudes in health care (DHCA)					
Disability session (*n* = 54)	68.9	68.9	*p* = 0.99	68.5	*p* = 0.57
Elective course (*n* = 8)	70.25	69.5	*p* = 0.75	71.4	*p* = 0.51
Session vs. Elective	*p* = 0.36	*p* = 0.8			
Jefferson scale of empathy (JSE-S)					
Disability session (*n* = 54)	120.3	120.3	*p* = 0.97	121.3	*p* = 0.38
Elective course (*n* = 8)	123.25	122.3	*p* = 0.63	125.3	*p* = 0.17
Session vs. Elective	*p* = 0.31	*p* = 0.63			
Anxiety Scale					
Disability session (*n* = 54)	56.1	54.8	*p* = 0.35	54.8	*p* = 0.95
Elective course (*n* = 8)	54.63	40.13	***p* = 0.1**	39.9	*p* = 0.53
Session vs. Elective	*p* = 0.64	***p* = 0.0007**		***p* = 0.01**	

Bolded *p*-values indicate statistical significance.

**Figure 4:**
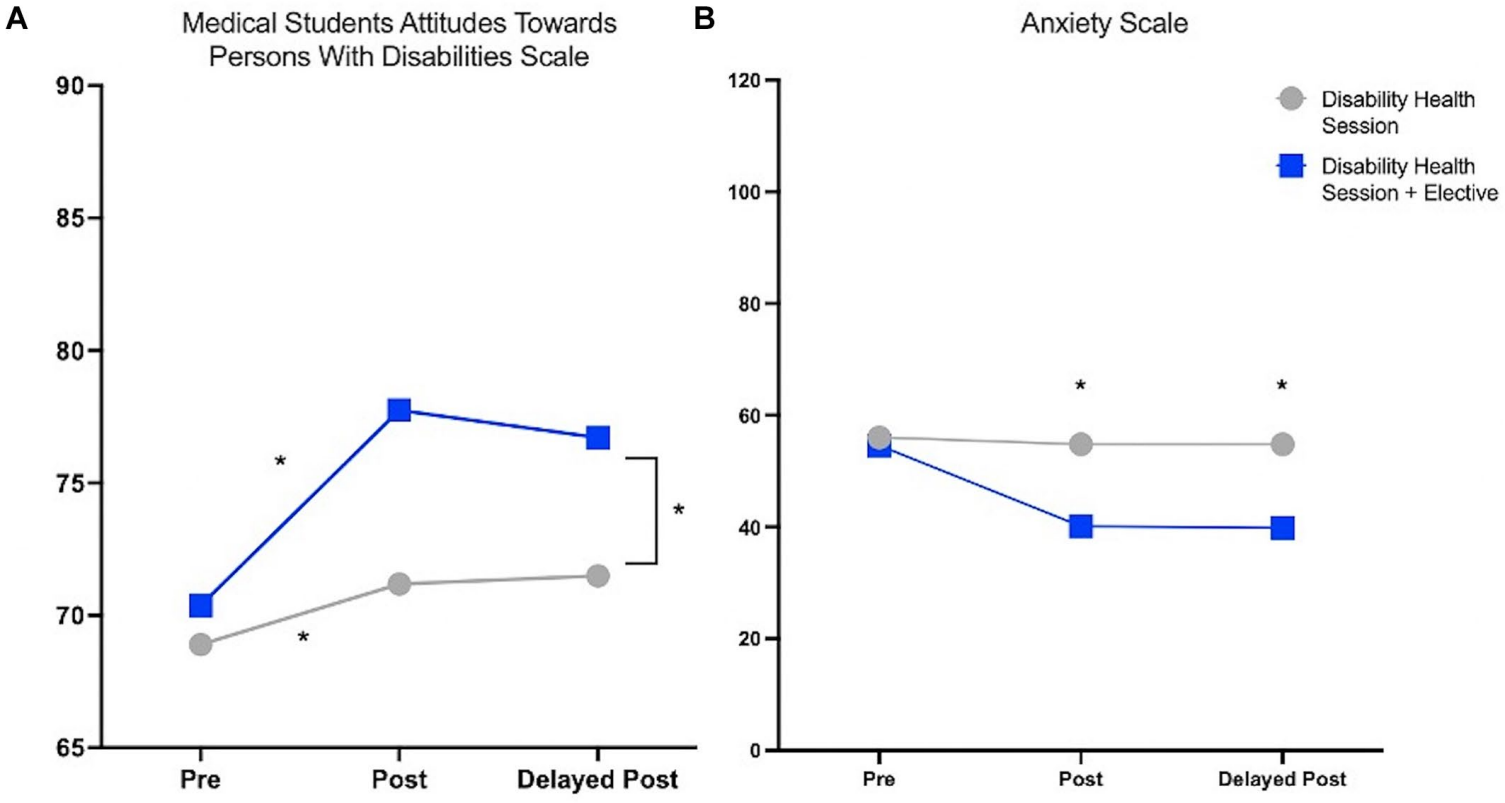
Comparing the Post Survey evaluations between the Disability health 2 h session versus Disability Elective measured by Medical Students Attitudes Toward Persons with Disabilities Scale and Anxiety scale. **(A)** Students who took the Disability health session and Disability elective had significantly higher scores on the Medical Students Attitudes Toward Persons with Disabilities scale (71.2 vs. 77.8, *p* = 0.006; Student’s *t*-test). Delayed post survey shows that the scores were maintained 3 months after the post survey was completed (71.5 vs. 76.9, *p* = 0.05). **(B)** Students who took the Disability health session and Disability elective had significantly lower scores on the Anxiety scale indicating lower anxiety (54.8 vs. 40.13, *p* = 0.0007; Student’s *t*-test). Delayed post-survey shows that the scores were maintained on the Anxiety scale 3 months after the course was completed (54.8 vs. 39.9, *p* = 0.01; Student’s *t*-test).

Overall, both interventions resulted in improvement in students’ attitudes toward individuals with disabilities. When comparing the students who received just the 2 h Disability Health session, with those who also took the Disability Elective course, there was a significant difference in post survey attitudes as reflected by the Medical Students Attitudes Toward Persons with Disabilities Scale (71.2 vs. 77.8, *p* = 0.006) (Figure 4A). In addition, anxiety was significantly reduced for students in the Disability elective compared to the students who only took the 2 h disability health session (54.8 vs. 40.13, *p* = 0.007) (Figure 4B). The differences in attitudes and anxiety were maintained on a repeat survey 3 months after the educational interventions were completed (Supplementary Figure S3A,B).

When looking at the individualized statements from the surveys, students who took the elective class had much less overall anxiety when caring for patients with disabilities (less uncertain, less worried, less threatened, less anxious and more at ease, more comfortable and safe; Supplementary Table S14). Students who took the elective course compared to students taking just the 2-h session felt more “comfortable adapting my body positions to facilitate effective communication for someone who is visually or hearing impaired” (4.11vs. 4.75, *p* = 0.045), more “confident in communicating with patients with disabilities” (3.26 vs. 4.25, *p* = 0.017), and more “confident in my understanding of cultural, economic and physical barriers to participation” (3.44 vs. 4.5, *p* = 0.007) (Supplementary Table S15). Students overall in the disability elective felt more “prepared to take care of patients with disabilities” (2.48 vs. 3.625, *p* = 0.005).

## Discussion

People with disabilities represent 26% of patients in the US, and yet many physicians feel inadequately equipped to care for this population. As a result, several national organizations have published calls to action to improve medical education surrounding caring for those with disabilities (21, 27). In this study, our objective was to evaluate the impact of two curricular interventions on medical students’ attitudes, empathy, and anxiety when caring for individuals with disabilities.

Our study demonstrated that the mandatory disability session improved student attitudes toward individuals with disabilities based on the Medical Student Attitudes Toward Persons with Disabilities survey (15). However, the total score increase was modest. While students reported that they felt more prepared after the mandatory disability session, the absolute values of the preparedness scores still showed that the average students still felt neutral or disagreed that they were prepared to care for patients with disabilities. The survey response indicates that while a 2 h session can improve self-reported preparedness, it is not sufficient for medical education on disability health. Importantly, although other disability education studies have not investigated the long term impact of interventions, our study noted that improved attitudes remained at the time of the delayed post-survey, 3 months after the intervention (27). Although our study showed there was benefit 3 months after the intervention, a recent study has shown that in another disability curricular intervention attitude and empathy gains 1 year later did not sustain the benefits (44). The 2 h curriculum did not reduce student’s anxiety levels toward caring for individuals with disabilities, which we hypothesize was due to a lack of direct interaction with this population. In both interventions, the students did not score differently on the Jefferson Scale of Empathy. One possible explanation is that the survey is not disability specific. Another potential rationale for this finding is that medical students in our study had a high baseline empathy score (mean = 120.3) compared to the JSE mean when it was created (mean = 114.3). This difference in baseline empathy scores may result from prior exposure to other modules in medical school curricula and through screening through the medical school’s admissions process, and as a result our intervention may have had a lower potential impact on JSE scores. In contrast to their improvement in attitudes on the Medical Student Attitudes Toward Persons with Disabilities survey in both intervention groups, there was no difference on the Disabilities Attitudes in Health Care scale. This difference could be due to the way the two tools were developed. The DAHC was adapted from two prior scales which were based on geriatric scales (39), whereas the MSATP was adapted from seven scales and incorporated significant input from the disability community including patients and families, medical educators, and local professionals who work with people with disabilities (15, 45). Although students’ attitudes and preparedness improved on the MSATP with the 2 h curricular intervention, the majority of students still disagreed with the statement that they felt prepared to care for patients with disabilities, which indicates although there was a statistical increase, there was likely a gap in clinical significance, arguing for the importance of more spaced repetition and integration of disability health throughout the longitudinal medical curriculum. When developing the disability elective course, we specifically designed it to include more direct interaction with individuals with disabilities and skills training surrounding the recommended core competencies with the hypothesis that it would have greater impact on attitudes and behavior (13, 37).

We demonstrate here that the 9 week elective course was an effective educational intervention. The students who took the elective course scored significantly higher on the Medical Students Attitudes Toward Persons with Disabilities Scale and lower on the Anxiety scale compared to the 2 h disability session alone. Their reduction on the Anxiety scale supports the intergroup contact hypothesis, where frequent and early interactions with individuals with disabilities in medical training improves comfort (15, 46–48). After the communications skills workshops, students felt more confident in communicating with patients with disabilities compared to their counterparts who only did the 2 h session. The ability to practice skills and receive feedback likely contributed to overall feeling prepared. Students who took the comprehensive elective course on average agreed that they were prepared to care for individuals with disabilities, unlike the students who just took the 2 h session.

Given the positive impact of our curriculum, we hope that all medical schools consider adopting similar sessions to improve disability competency. We show here that even a short 2-h disability session can improve medical student attitudes toward patients with disabilities, however, is not sufficient to help students feel prepared. The elective could be taught as a stand-alone course as is the trend among other medical schools with the development of disability health electives (32) or components of our elective session could be included throughout required medical school training (23) and be utilized to modify core EPAs to cover competencies (13). Further research would include incorporating components of the disability elective curriculum into the mandatory curriculum and evaluating students throughout different stages of their medical education.

Given the challenge of limited time to add additional sessions into medical school curricula, we propose ways to include components of our elective course into existing parts of standard medical school didactics. Didactic sessions from the elective which include the history of disability and the ethics of disability, and teaching on the social model of disability could be converted into online modules for ease of student accessibility to the information. Additionally, during practicum skills sessions, individuals with disabilities should be included during history and physical exam teaching sessions, as it has been shown that students do not perform as well on practical skills exams with individuals with disabilities if they have not received specific practice prior (49). Educators at medical schools have expressed difficulty in incorporating people with disabilities as teachers, however, we found in development of our course that there are many existing local and national organizations that medical schools can partner with to gain access to disability educators.

Our study differs from other published brief disability curricular interventions in that we uniquely utilized members of the healthcare professions with disabilities to participate in the patient panel and in the discussion groups, in order to normalize disability as diversity in the medical profession. A study at Stanford that showed 28.4% of faculty, students, and staff in the School of Medicine reported having an ADA defined disability. However, public self-identification of disability within the institution is rare for individuals with invisible disabilities (50). Our educational interventions also included participants with a wide range of disabilities, showcasing the diversity within disability.

This study was not without significant limitations. Overall, the study would have vastly benefited from a greater sample size, as the numbers in this study were very modest, and randomization of participants into the different interventions, to try and reduce the bias of students with greater interest taking the elective course. The study design would have strongly benefited from a control group from a different institution who did not have any disability curriculum at the time of intervention. Since students chose to take the longer elective course, there could be bias between the two groups in their motivation to learn about patients with disabilities. People in the elective course had more career interest in working with individuals with disabilities, which could explain the trend of slightly more positive attitudes on the pre-survey MSATPD scale (70.4) compared to the other group (68.4), although this was not statistically significant. Low response rates and participant retention is a complication of web-based data collection, and the sample of participants in this study may not be entirely representative of the general medical student population. Generalizability of the finding is also limited by the location of the study at only one medical school. Additionally, we recognize that survey measures used in this study are not direct proxies for providers’ actions. In the future, studies comparing students’ self-rated scores to patient’s perceptions of their care, and overall patient outcomes would be more direct measures of the effect of our interventions. In light of limitations, the study strengths included a multi-disciplinary team approach from multiple perspectives in medicine. Additionally, our surveys were anonymous, minimizing social desirability bias.

## Conclusion

Our findings provide support that both a brief 2 h curricular intervention and a more comprehensive 9 week elective curriculum can improve medical students’ attitudes toward individuals with disabilities with a long term effect. In comparing the two interventions, we highlight the importance of direct interaction with individuals with disabilities as specific communications workshops, panels, and partner programs further reduced anxiety and better prepared medical students to care for individuals with disabilities. We hope that similar curriculums can be incorporated at medical schools across the country. Reducing healthcare disparities toward individuals with disabilities will require a multifaceted approach with system wide changes in our healthcare system, and it is essential that medical students have exposure to individuals with disabilities as a basic tenet to improve healthcare for this population.

## Data Availability

The original contributions presented in the study are included in the article/Supplementary material, further inquiries can be directed to the corresponding author.

## Supplementary material

SUPPLEMENTARY DATA SHEET 1 Supplemental Figures and Tables.

SUPPLEMENTARY DATA SHEET 2 Supplemental Appendix 1.
