# Supplementary material for: Disability health in medical education: development, implementation, and evaluation of a pilot curriculum at Stanford School of Medicine
Source: Front Med (Lausanne). 2024 Sep 4;11:1355473. doi: 10.3389/fmed.2024.1355473 (PMC11408233; doi:10.3389/fmed.2024.1355473)
Supplement: SUPPLEMENTARY DATA SHEET 1 — Supplemental Figures and Tables. [file Data_Sheet_1.DOCX]

Supplemental Figure 1


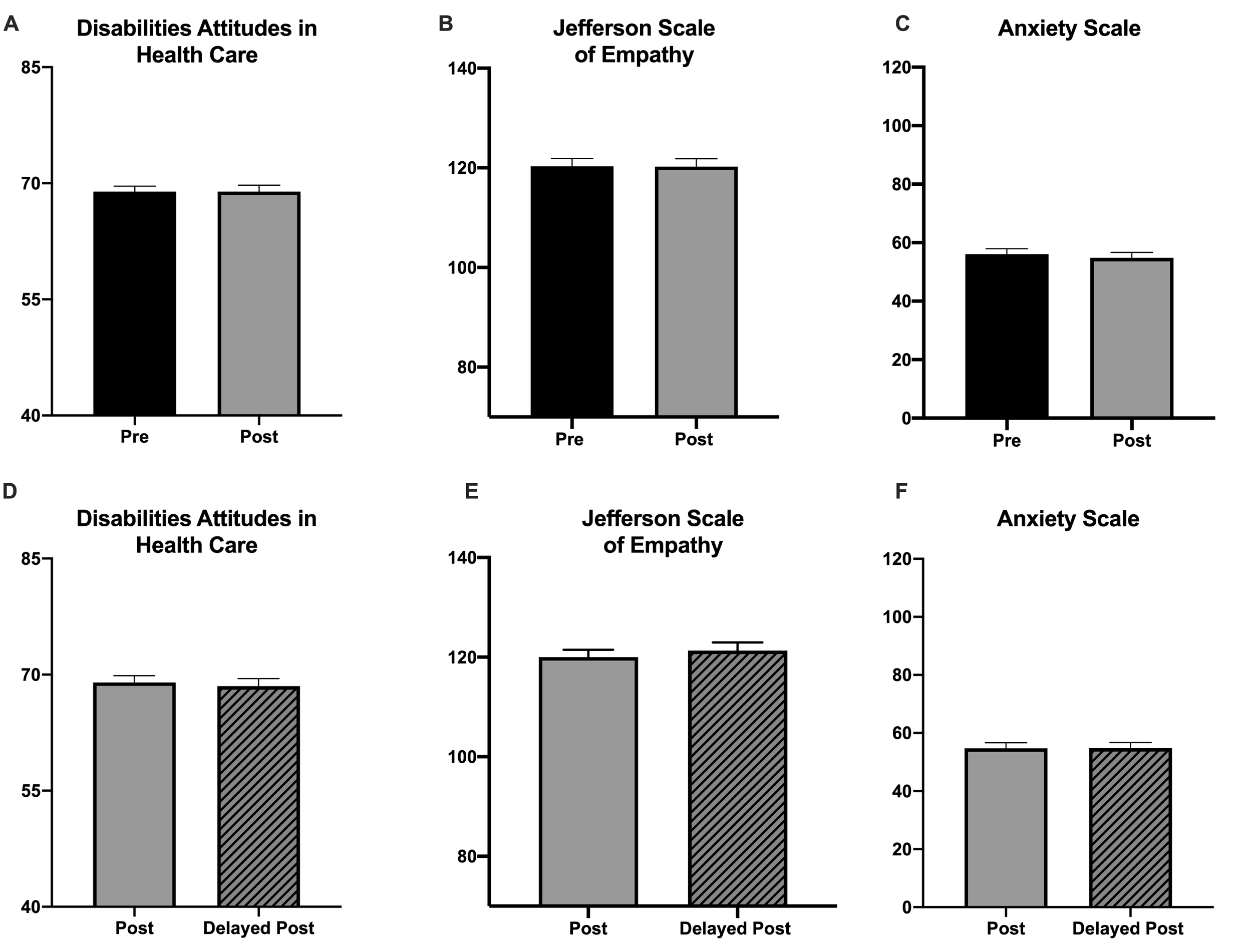


**A, B, C:** There were no significant differences comparing the pre and post test in the Disability Attitudes in Health Care Scale (DAHC) (68.9 vs 68.9, n =54, p=0.99), Jefferson Scale of Empathy (120.3 vs 120.3, n=54, p=0.97), or Anxiety Scale (56.1 vs 54.8, n=54, p=0.35) scores by Students paired t-test **D, E, F:** There were no significant differences comparing the post and delayed post in the Disability Attitudes in Health Care Scale (DAHC) (69 vs 68.5, n=46, p=0.57), Jefferson Scale of Empathy (120. vs 121.3, n=46, p=0.38), or Anxiety Scale (54.7 vs 54.8, n=46, p=0.95) by Student's paired t-test.

Supplemental Figure 2


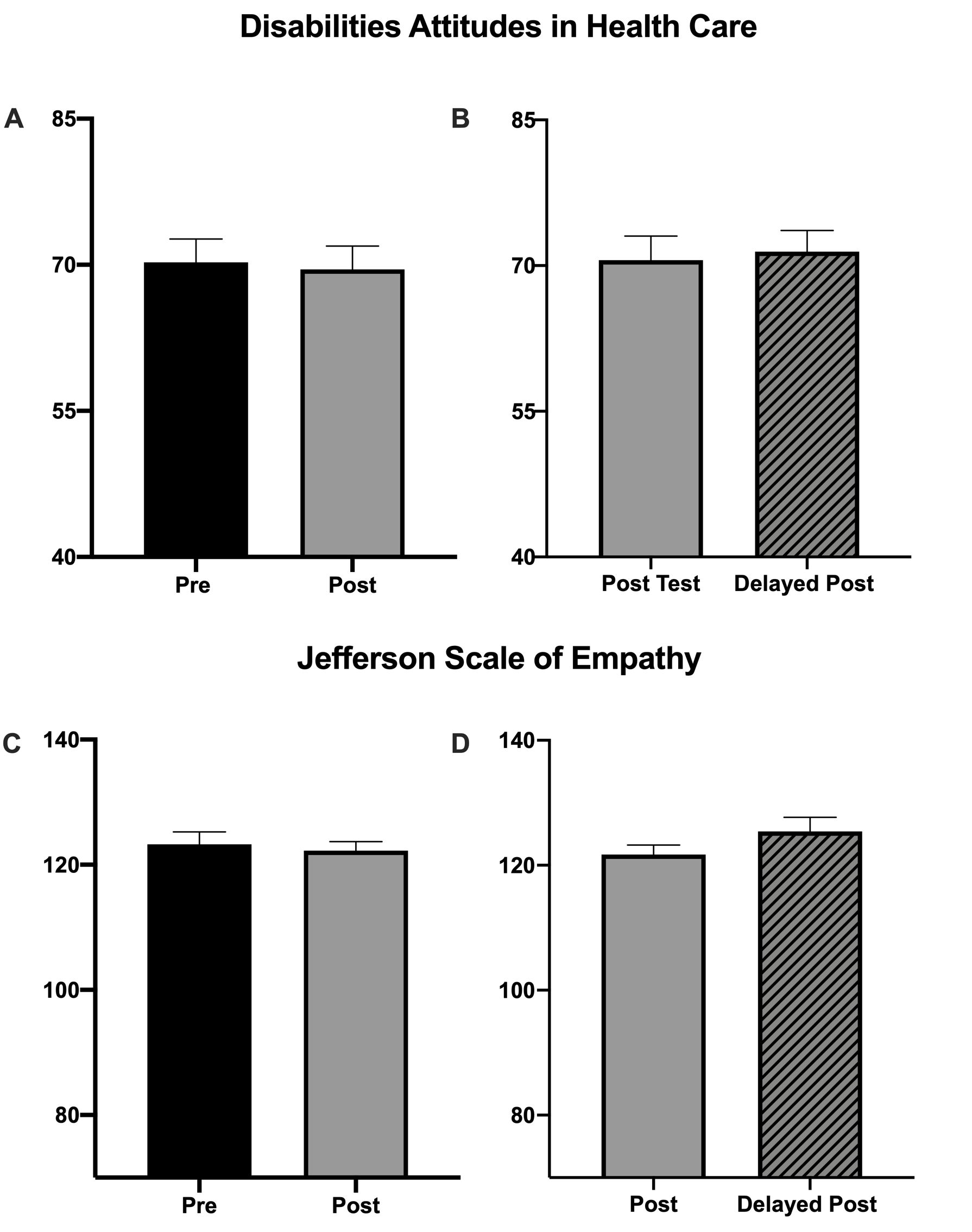


**A, B:** There were no significant differences comparing the pre and post test in the Disability Attitudes in Health Care Scale (DAHC) (70.25 vs 69.5, n =8, p=0.75) and the Jefferson Scale of Empathy (JSE) (123.25 vs 122.3, n=8, p=0.63) by Student's paired t-test. **C, D:** There was no significant difference comparing the post test and delayed post test in the DAHC (70.6 vs. 71.4, n=7, p=0.51) or JSE (121.7 vs 125.3, n=7, p=0.17) by Student's paired t-test.

Supplemental Table 1

| Item Scores on the Medical Student Attitudes Towards Persons with Disabilities for the Pre-, Post and Delayed Post for the Disability Health Session |  |  |  |  |  |
| --- | --- | --- | --- | --- | --- |
| Please choose the appropriate answer choice which best corresponds with how you feel about the statement. Choose only ONE response for each question. | Pre-survey | Post-survey | Pᵃ (pre vs post) | Delayed Post-survey | Pᵃ (post vs delayed post) |
| 1. Most people with disabilities feel sorry for themselves.ᵇ | 3.130 | 3.315 | **0.049** | 3.327 | 0.212 |
| 2. I am comfortable being around a person who has an intellectual disability | 3.074 | 2.981 | 0.229 | 2.959 | 0.444 |
| 3. People with disabilities are as happy as people without disabilities. | 2.963 | 3.204 | **0.006** | 3.204 | 0.518 |
| 4. I would be comfortable interacting with a person with an intellectual disability who was in the community on his or her own (i.e, without staff members or caretakers). | 3.000 | 3.093 | 0.389 | 3.041 | 0.837 |
| 5. I would be comfortable being around a person who uses a wheelchair. | 3.630 | 3.556 | 0.289 | 3.531 | 0.642 |
| 6. Most people with disabilities resent people without disabilities.ᵇ | 3.241 | 3.519 | **0.001** | 3.531 | 0.322 |
| 7. I would be comfortable being around a person who is deaf. | 3.185 | 3.167 | 0.837 | 3.184 | 0.850 |
| 8. I am only comfortable around people with intellectual disabilities if they are well-behaved.ᵇ | 2.796 | 2.704 | 0.374 | 2.735 | 0.743 |
| 9. Most people with disabilities expect special treatment.ᵇ | 3.185 | 3.167 | 0.821 | 3.224 | 0.420 |
| 10. I would be comfortable working with a person with an intellectual disability who had someone assigned to supervise or train her. | 3.241 | 3.333 | 0.279 | 3.306 | 0.200 |
| 11. Most people with disabilities are not ashamed of their disability. | 2.963 | 3.111 | 0.059 | 3.102 | **0.019** |
| 12. If I were visited by a person who is blind, I would be comfortable helping him or her navigate the environment. | 3.167 | 3.333 | 0.129 | 3.306 | 0.375 |
| 13. I am more comfortable around people with intellectual disabilities when they have someone who is not disabled to help them.ᵇ | 2.278 | 2.185 | 0.341 | 2.163 | 0.537 |
| 15. People with disabilities should be cared for in any primary care office as opposed to a specialty clinic. | 2.796 | 2.833 | 0.719 | 2.857 | 0.181 |
| 16. I would feel comfortable living next door to a person with an intellectual disability that lives by himself. | 3.130 | 3.259 | 0.196 | 3.265 | 0.569 |
| 17. I would be comfortable living in a neighborhood where there is a group home for people with various developmental disabilities (e.g., Down Syndrome, Cerebral Palsy) | 3.407 | 3.481 | 0.322 | 3.490 | 0.444 |
| 18. I would feel comfortable being around a person with an intellectual disability in public even though his behavior might be a bit bizarre (e.g., rocking back and forth, talking loud, etc.). | 3.000 | 3.019 | 0.799 | 3.000 | 1.000 |
| Scenario A:  You enter the exam room. A middle-aged man and woman are there. He tells you he is experiencing chronic abdominal pain. |  |  |  |  |  |
| 20. In scenario A, I would be comfortable determining the role of the man vs. the woman in providing the history of the complaint. | 3.148 | 3.389 | **0.018** | 3.327 | 0.533 |
| 21. In scenario A, I would be comfortable performing a physical exam on the patient. | 3.185 | 3.370 | 0.096 | 3.388 | 0.569 |
| 22. In scenario A, I would be comfortable establishing a differential diagnosis for the abdominal pain. | 2.667 | 3.111 | **0.001** | 3.061 | 0.785 |
| Scenario B:  You enter the exam room. A middle-aged man is seated in a wheelchair. Standing behind him is a woman of about the same age. The patient in the wheel chair appears to have spasticity in all 4 limbs. He greets you by saying "hello". His speech is somewhat garbled, though intelligible. The woman tells you that the patient is here because he is experiencing chronic abdominal pain. |  |  |  |  |  |
| 24. In scenario B, I would be comfortable determining the role of the man vs. the woman in providing the history of the complaint. | 2.852 | 3.093 | **0.031** | 3.102 | 1.000 |
| 25. In scenario B, I would be comfortable performing a physical exam on the patient. | 2.500 | 2.426 | 0.551 | 2.327 | 0.224 |
| 26. In scenario B, I would be comfortable establishing a differential diagnosis for the abdominal pain. | 2.370 | 2.537 | 0.211 | 2.469 | **0.024** |
| ᵃPaired T-test |  |  |  |  |  |
| ᵇThe scores for negatively worded items were inverted so that higher scores indicate stronger disagreement toward these statements. |  |  |  |  |  |
| Boldface indicates statistical significance. |  |  |  |  |  |

Supplemental Table 2:

| Item Scores on the Disability Attitudes In Healthcare Survey for the Pre-, Post and Delayed Post for the Disability Health Session |  |  |  |  |  |
| --- | --- | --- | --- | --- | --- |
| Please use the scale to indicate the degree to which you agree or disagree with each statement. There are no right or wrong answers. The best response is the one that truly reflects your personal opinion. | Pre-survey | Post-survey | Pᵃ (pre vs post) | Delayed Post-survey | Pᵃ (post vs delayed post) |
| 1. People with disabilities are pleasant to be with. | 4.056 | 4.167 | 0.243 | 3.957 | 0.185 |
| 2. If I had a choice, I would rather see able-bodied patients than patients with disabilities.ᵇ | 3.296 | 3.315 | 0.890 | 3.087 | 0.173 |
| 3. It is society's responsibility to provide care for its persons with disabilities. | 4.519 | 4.463 | 0.569 | 4.283 | 0.058 |
| 4. Patients with disabilities improve with treatment. | 3.648 | 3.685 | 0.784 | 3.674 | 0.660 |
| 5. Medical care for PWDs uses up too many resources.ᵇ | 4.519 | 4.333 | 0.510 | 4.304 | 0.439 |
| 6. Taking a medical history from a patient with disability is frequently an ordeal.ᵇ | 3.370 | 3.481 | 0.428 | 3.587 | 0.499 |
| 7. People with disability, in general, do not contribute much to society.ᵇ | 4.667 | 4.519 | 0.172 | 4.543 | 0.728 |
| 8. People with disability don't contribute their fair share toward paying for their health care.ᵇ | 4.204 | 4.222 | 0.883 | 4.222 | 1.000 |
| 9. I will welcome patients with disabilities into my practice. | 4.815 | 4.667 | 0.053 | 4.667 | 1.000 |
| 10. If handled properly, the patient with disability can be seen as quickly as any other patient. | 3.481 | 3.722 | 0.129 | 3.739 | 0.522 |
| 11. Understanding my patients with disabilities is valuable to me as a physician. | 4.889 | 4.741 | 0.063 | 4.696 | 0.497 |
| 12. Patients with disability are better off in nursing homes.ᵇ | 4.463 | 4.537 | 0.376 | 4.565 | 0.622 |
| 13. Medicare offers adequate compensation for care of patients with disability. | 2.278 | 2.278 | 1.000 | 2.152 | 0.481 |
| 14. The treatment of patients with disability is too time consuming.ᵇ | 3.963 | 3.944 | 0.864 | 4.043 | 0.429 |
| 15. More training is needed to prepare health practitioners to provide care to the patient with disability. | 4.630 | 4.685 | 0.444 | 4.696 | 0.799 |
| 16. It is unglamourous to care for people with disability.ᵇ | 3.370 | 3.426 | 0.745 | 3.478 | 0.803 |
| 17. Treatment of people with disability is hopeless.ᵇ | 4.759 | 4.741 | 0.844 | 4.741 | 1.000 |

ᵃPaired T-test

ᵇThe scores for negatively worded items were inverted so that higher scores indicate stronger disagreement toward these statements.

Boldface indicates statistical significance.

Supplemental Table 3

| Item Scores on the Jefferson Scale of Empathy for the Pre-, Post and Delayed Post for the Disability Health Session |  |  |  |  |  |
| --- | --- | --- | --- | --- | --- |
| Please use the following 7-point scale (a higher number on the scale indicates more agreement): Mark one and only one response for each statement | Pre-survey | Post-survey | Pᵃ (pre vs post) | Delayed Post-survey | Pᵃ (post vs delayed post) |
| 1. Physicians' understanding of their patients' feelings and the feelings of their patients' families does not influence medical or surgical treatment.ᵇ | 6.02 | 5.98 | 0.83 | 6.13 | 0.30 |
| 2. Patients feel better when their physicians understand their feelings. | 6.67 | 6.74 | 0.62 | 6.81 | 0.57 |
| 3. It is difficult for physician to view things from patients' perspectives. | 4.69 | 4.91 | 0.33 | 5.32 | 0.12 |
| 4. Understanding body language is as important as verbal communication in physician-patient relationships. | 6.26 | 6.20 | 0.70 | 6.21 | 0.75 |
| 5. A physician's sense of humor contributes to a better clinical outcome. | 5.17 | 5.31 | 0.39 | 5.47 | 0.28 |
| 6. Because people are different, it is difficult to see things from patients' perspectives.ᵇ | 4.76 | 4.78 | 0.93 | 5.23 | 0.04 |
| 7. Attention to patients' emotions is not important in history taking.ᵇ | 6.65 | 6.26 | 0.09 | 6.55 | 0.21 |
| 8. Attentiveness to patients' personal experiences does not influence treatment outcomes.ᵇ | 6.41 | 6.33 | 0.58 | 6.40 | 0.34 |
| 9. Physicians should try to stand in their patients' shoes when providing care to them. | 6.13 | 6.07 | 0.69 | 6.02 | 0.89 |
| 10. Patients value a physician's understanding of their feelings which is therapeutic in its own right. | 6.13 | 6.17 | 0.79 | 6.32 | 0.19 |
| 11. Patients' illnesses can be cured only by medical or surgical treatment; therefore, physicians' emotional ties with their parents do not have a significant influence in medical or surgical treatment.ᵇ | 6.46 | 6.50 | 0.78 | 6.38 | 0.32 |
| 12. Asking patients about what is happening in their personal lives is not helpful in understanding their physical complaints.ᵇ | 6.46 | 6.52 | 0.77 | 6.47 | 0.69 |
| 13. Physicians should try to understand what is going on in their patients' minds by paying attention to their non-verbal cues and body language. | 6.35 | 6.06 | 0.11 | 6.15 | 0.61 |
| 14. I believe that emotion has no place in the treatment of medical illness.ᵇ | 6.65 | 6.61 | 0.83 | 6.64 | 0.85 |
| 15. Empathy is a therapeutic skill without which the physician's success is limited. | 6.35 | 6.13 | 0.15 | 6.19 | 0.71 |
| 16. Physicians' understanding of the emotional status of their patients, as well as that of their families is one important component of the physician-patient relationship. | 6.46 | 6.43 | 0.78 | 6.41 | 0.87 |
| 17. Physicians should try to think like their patients in order to render better care. | 5.69 | 5.87 | 0.25 | 5.81 | 0.92 |
| 18. Physicians should not allow themselves to be influenced by strong personal bonds between their patients and their family members.ᵇ | 4.41 | 4.57 | 0.44 | 4.36 | 0.80 |
| 19. I do not enjoy reading non-medical literature or the arts.ᵇ | 6.20 | 6.28 | 0.67 | 6.17 | 0.29 |
| 20. I believe that empathy is an important therapeutic factor in medical treatment. | 6.41 | 6.54 | 0.38 | 6.40 | 0.40 |

ᵃPaired T-test

ᵇThe scores for negatively worded items were inverted so that higher scores indicate stronger disagreement toward these statements.

Boldface indicates statistical significance.

Supplemental Table 4

| Item Scores on the Anxiety Scale for the Pre-, Post and Delayed Post for the Disability Health Session |  |  |  |  |  |
| --- | --- | --- | --- | --- | --- |
| Please for each of the items listed below, indicate how you feel when interacting with individuals with disabilities. | Pre-survey | Post-survey | Pᵃ (pre vs post) | Delayed Post-survey | Pᵃ (post vs delayed post) |
| 1. Nervous | 5.39 | 5.19 | 0.46 | 5.11 | 0.84 |
| 2. Friendlyᵇ | 2.78 | 2.87 | 0.72 | 2.83 | 0.76 |
| 3. Uncertain | 6.09 | 6.00 | 0.70 | 5.98 | 0.86 |
| 4. Comfortableᵇ | 5.00 | 5.28 | 0.23 | 4.83 | 0.14 |
| 5. Worried | 4.91 | 4.59 | 0.24 | 4.61 | 0.70 |
| 6. Trustingᵇ | 3.69 | 3.83 | 0.51 | 3.83 | 0.62 |
| 7. Threatened | 2.22 | 2.17 | 0.78 | 2.22 | 0.53 |
| 8. Confidentᵇ | 5.87 | 5.56 | 0.19 | 5.52 | 0.78 |
| 9. Awkward | 5.80 | 5.56 | 0.34 | 5.87 | 0.47 |
| 10. Safeᵇ | 3.54 | 3.19 | 0.17 | 3.15 | 0.64 |
| 11. Anxious | 5.04 | 4.98 | 0.83 | 5.11 | 0.70 |
| 12. At Easeᵇ | 5.74 | 5.61 | 0.53 | 5.78 | 0.48 |
| ᵃPaired T-test |  |  |  |  |  |
| ᵇQuestions were inverted for scoring. |  |  |  |  |  |
| Boldface indicates statistical significance. |  |  |  |  |  |

Supplemental Table 5:

| Item Scores on Comfort and Confidence Scales for the Pre-, Post and Delayed Post for the Disability Health Session |  |  |  |  |  |
| --- | --- | --- | --- | --- | --- |
| Please use the scale to indicate the degree to which you agree or disagree with each statement. There are no right or wrong answers. The best response is the one that truly reflects your personal opinion. | Pre-survey | Post-survey | Pᵃ (pre vs post) | Delayed Post-survey | Pᵃ (post vs delayed post) |
| 1. I feel comfortable approaching someone with a disability who may need assistance.ᵇ | 4.092593 | 4.166667 | 0.532112 | 4.166667 | 1.000000 |
| 2. I am comfortable providing assistance appropriately to a person with a disability.ᵇ | 3.388889 | 3.703704 | **0.048659** | 3.702128 | 0.755560 |
| 3. I am comfortable adapting my body positions to make someone who uses a wheelchair more comfortable.ᵇ | 4.000000 | 3.888889 | 0.517761 | 3.914894 | 0.805630 |
| 4. I am comfortable adapting my body positions to facilitate effective communication for someone who is visually or hearing impaired.ᵇ | 3.962963 | 4.111111 | 0.289250 | 4.191489 | 0.854996 |
| 5. I am comfortable adapting my interviewing technique to accommodate patients with disabilities.ᵇ | 3.500000 | 3.814815 | 0.054964 | 3.872340 | 0.569293 |
| 6. I am comfortable using appropriate communication techniques to gather accurate and complete information from patients who may be non-verbal.ᵇ | 2.777778 | 3.129630 | 0.091990 | 3.042553 | 0.729559 |
| 7. I am comfortable adapting physical examination techniques to accommodate persons with disabilities.ᵇ | 2.870370 | 3.000000 | 0.545776 | 3.042553 | 0.816549 |
| 8. I can see myself as an advocate for my patients with disabilities.ᵇ | 4.166667 | 4.000000 | 0.129389 | 3.957447 | 0.552181 |
| 9. I feel prepared to take care of patients with disabilities.ᶜ | 2.074074 | 2.481481 | **0.013689** | 2.744681 | 0.175072 |
| 10. I feel confident in communicating with patients with disabilities.ᶜ | 2.685185 | 3.259259 | **0.000724** | 3.446809 | 0.150901 |
| 11. I feel confident in my understanding of "disability".ᶜ | 3.129630 | 3.907407 | **0.000000** | 4.148936 | 0.041981 |
| 12. I feel confident in knowledge of barriers to access to care for persons with disabilities.ᶜ | 2.722222 | 3.555556 | **0.000000** | 3.829787 | 0.033280 |
| 13. I feel confident in my understanding of the concept of ability and participation in the International Classification of Function.ᶜ | 2.000000 | 2.685185 | **0.000098** | 2.765957 | 0.523451 |
| 14. I feel confident in my understanding of cultural, economic, and physical barriers to participation.ᶜ | 2.703704 | 3.444444 | **0.000008** | 3.595745 | 0.281016 |
| 15. I feel confident in knowledge about my understanding about the quality of life issues for people with disability.ᶜ | 2.648148 | 3.407407 | **0.000002** | 3.617021 | 0.035900 |
| ᵃPaired T-test |  |  |  |  |  |
| ᵇAdapted from a Survey Scale section ‘Interacting with the Disabled” and one question from the “Advocacy” scale (Saketkoo et al. 2004). |  |  |  |  |  |
| ᶜNewly developed confidence questions by medical education experts |  |  |  |  |  |
| Boldface indicates statistical significance. |  |  |  |  |  |

Supplemental Table 6:

| Item Scores on the Medical Student Attitudes Towards Persons with Disabilities for the Pre-, Post and Delayed Post for the Disability Elective |  |  |  |  |  |
| --- | --- | --- | --- | --- | --- |
| Please choose the appropriate answer choice which best corresponds with how you feel about the statement. Choose only ONE response for each question. | Pre-survey | Post-survey | Pᵃ (pre vs post) | Delayed Post-survey | Pᵃ (post vs delayed post) |
| 1. Most people with disabilities feel sorry for themselves.ᵇ | 3.5 | 3.75 | 0.3506166628 | 3.75 | 1 |
| 2. I am comfortable being around a person who has an intellectual disability | 3.125 | 3.375 | 0.1704706608 | 3.375 | 1 |
| 3. People with disabilities are as happy as people without disabilities. | 3.375 | 3.375 | 1 | 3.375 | 1 |
| 4. I would be comfortable interacting with a person with an intellectual disability who was in the community on his or her own (i.e, without staff members or caretakers). | 2.875 | 3.625 | **0.04794477218** | 3.142857143 | 0.3559176837 |
| 5. I would be comfortable being around a person who uses a wheelchair. | 3.375 | 3.625 | 0.3506166628 | 3.428571429 | 0.1723082967 |
| 6. Most people with disabilities resent people without disabilities.ᵇ | 3.375 | 3.75 | 0.07960201246 | 3.75 | 1 |
| 7. I would be comfortable being around a person who is deaf. | 3.125 | 3.5 | 0.2849313808 | 3.714285714 | 0.1723082967 |
| 8. I am only comfortable around people with intellectual disabilities if they are well-behaved.ᵇ | 3 | 2.75 | 0.5630278037 | 3.285714286 | 0.1403463009 |
| 9. Most people with disabilities expect special treatment.ᵇ | 3.625 | 3.625 | 1 | 3.285714286 | 0.1723082967 |
| 10. I would be comfortable working with a person with an intellectual disability who had someone assigned to supervise or train her. | 3.375 | 3.5 | 0.598331156 | 3.428571429 | 0.6036450565 |
| 11. Most people with disabilities are not ashamed of their disability. | 2.875 | 3.5 | 0.09497649244 | 3.428571429 | 0.3559176837 |
| 12. If I were visited by a person who is blind, I would be comfortable helping him or her navigate the environment. | 2.875 | 3.25 | 0.1970220722 | 3 | 0.3559176837 |
| 13. I am more comfortable around people with intellectual disabilities when they have someone who is not disabled to help them.ᵇ | 1.75 | 2 | 0.3506166628 | 2.428571429 | 0.07814074941 |
| 15. People with disabilities should be cared for in any primary care office as opposed to a specialty clinic. | 3 | 3 | 1 | 3.142857143 | 0.6890522264 |
| 16. I would feel comfortable living next door to a person with an intellectual disability that lives by himself. | 3.25 | 3.625 | 0.07960201246 | 3.625 | 1 |
| 17. I would be comfortable living in a neighborhood where there is a group home for people with various developmental disabilities (e.g., Down Syndrome, Cerebral Palsy) | 3.625 | 3.875 | 0.1704706608 | 3.714285714 | 0.3559176837 |
| 18. I would feel comfortable being around a person with an intellectual disability in public even though his behavior might be a bit bizarre (e.g., rocking back and forth, talking loud, etc.). | 3.375 | 3.375 | 1 | 3.571428571 | 0.3559176837 |
| Scenario A:  You enter the exam room. A middle-aged man and woman are there. He tells you he is experiencing chronic abdominal pain. |  |  |  |  |  |
| 20. In scenario A, I would be comfortable determining the role of the man vs. the woman in providing the history of the complaint. | 2 | 3.75 | 0.1970220722 | 3.75 | 1 |
| 21. In scenario A, I would be comfortable performing a physical exam on the patient. | 3 | 3.625 | 0.1704706608 | 3.714285714 | 0.3559176837 |
| 22. In scenario A, I would be comfortable establishing a differential diagnosis for the abdominal pain. | 1 | 3.5 | **0.03314550026** | 3.285714286 | 0.3559176837 |
| Scenario B:  You enter the exam room. A middle-aged man is seated in a wheel chair. Standing behind him is a woman of about the same age. The patient in the wheel chair appears to have spasticity in all 4 limbs. He greets you by saying "hello". His speech is somewhat garbled, though intelligible. The woman tells you that the patient is here because he is experiencing chronic abdominal pain. |  |  |  |  |  |
| 24. In scenario B, I would be comfortable determining the role of the man vs. the woman in providing the history of the complaint. | 2 | 3.625 | **0.01845152851** | 3.428571429 | 0.3559176837 |
| 25. In scenario B, I would be comfortable performing a physical exam on the patient. | 2 | 2.75 | 0.1395195831 | 2.571428571 | 0.6036450565 |
| 26. In scenario B, I would be comfortable establishing a differential diagnosis for the abdominal pain. | 1 | 3 | **0.0491737142** | 2.571428571 | 0.1723082967 |
| ᵃPaired T-test |  |  |  |  |  |
| ᵇThe scores for negatively worded items were inverted so that higher scores indicate stronger disagreement toward these statements. |  |  |  |  |  |
| Boldface indicates statistical significance. |  |  |  |  |  |

Supplemental Table 7:

| Item Scores on the Disability Attitudes In Healthcare Survey for the Pre-, Post and Delayed Post for the Disability Elective |  |  |  |  |  |
| --- | --- | --- | --- | --- | --- |
| Please use the scale to indicate the degree to which you agree or disagree with each statement. There are no right or wrong answers. The best response is the one that truly reflects your personal opinion. | Pre-survey | Post-survey | Pᵃ (pre vs post) | Delayed Post-survey | Pᵃ (post vs delayed post) |
| 1. People with disabilities are pleasant to be with. | 4.375 | 4.625 | 0.5164895523 | 4.285714286 | 0.1996216727 |
| 2. If I had a choice, I would rather see able-bodied patients than patients with disabilities.ᵇ | 3.5 | 3.875 | 0.3506166628 | 3.428571429 | 0.2308094088 |
| 3. It is society's responsibility to provide care for its persons with disabilities. | 4.625 | 4.375 | 0.3506166628 | 5 | 0.1723082967 |
| 4. Patients with disability improve with treatment. | 4.25 | 4.375 | 0.6845283356 | 4.714285714 | 0.1723082967 |
| 5. Medical care for PWDs uses up too many resources.ᵇ | 4.625 | 4.625 | 1 | 4.428571429 | 0.6036450565 |
| 6. Taking a medical history from a patient with disability is frequently an ordeal.ᵇ | 3.625 | 3.875 | 0.6845283356 | 4.142857143 | 0.1996216727 |
| 7. People with disability, in general, do not contribute much to society.ᵇ | 4.875 | 4.75 | 0.598331156 | 4.75 | 1 |
| 8. People with disability don't contribute their fair share toward paying for their health care.ᵇ | 4 | 4.375 | 0.4757972385 | 4.714285714 | 0.3559176837 |
| 9. I will welcome patients with disability into my practice. | 4.875 | 4.75 | 0.3506166628 | 4.75 | 1 |
| 10. If handled properly, the patient with disability can be seen as quickly as any other patient. | 3.125 | 2.75 | 0.2849313808 | 2.857142857 | 0.7357648599 |
| 11. Understand my patients with disability is valuable to me as a physician. | 4.625 | 4.75 | 0.3506166628 | 4.75 | 1 |
| 12. Patients with disability are better off in nursing homes.ᵇ | 4.625 | 4.625 | 1 | 4.857142857 | 0.1723082967 |
| 13. Medicare offers adequate compensation for care of patients with disability. | 2.125 | 1.25 | **0.006197520581** | 1.25 | 1 |
| 14. The treatment of patients with disability is too time consuming.ᵇ | 4.625 | 4.25 | 0.4015076246 | 4.25 | 1 |
| 15. More training is needed to prepare health practitioners to provide care to the patient with disability. | 4.875 | 4.875 | 1 | 4.875 | 1 |
| 16. It is unglamourous to care for people with disability.ᵇ | 2.5 | 2.375 | 0.7317884934 | 3.142857143 | 0.3208080965 |
| 17. Treatment of people with disability is hopeless.ᵇ | 5 | 5 | 1 | 4.857142857 | 0.3559176837 |
| ᵃPaired T-test |  |  |  |  |  |
| ᵇThe scores for negatively worded items were inverted so that higher scores indicate stronger disagreement toward these statements. |  |  |  |  |  |
| Boldface indicates statistical significance. |  |  |  |  |  |

Supplemental Table 8:

| Item Scores on the Jefferson Scale of Empathy for the Pre-, Post and Delayed Post for the Disability Elective |  |  |  |  |  |
| --- | --- | --- | --- | --- | --- |
| Please use the following 7-point scale (a higher number on the scale indicates more agreement): Mark one and only one response for each statement | Pre-survey | Post-survey | Pᵃ (pre vs post) | Delayed Post-survey | Pᵃ (post vs delayed post) |
| 1. Physicians' understanding of their patients' feelings and the feelings of their patients' families does not influence medical or surgical treatment.ᵇ | 6.88 | 6.13 | 0.08 | 6.86 | 0.05 |
| 2. Patients feel better when their physicians understand their feelings. | 6.63 | 6.88 | 0.35 | 6.71 | 0.17 |
| 3. It is difficult for physician to view things from patients' perspectives. | 3.88 | 4.38 | 0.35 | 5.29 | 0.08 |
| 4. Understanding body language is as important as verbal communication in physician-patient relationships. | 6.38 | 6.38 | 1.00 | 6.57 | 0.36 |
| 5. A physician's sense of humor contributes to a better clinical outcome. | 4.63 | 4.63 | 1.00 | 4.57 | 0.69 |
| 6. Because people are different, it is difficult to see things from patients' perspectives.ᵇ | 4.38 | 4.88 | 0.23 | 5.43 | 0.36 |
| 7. Attention to patients' emotions is not important in history taking.ᵇ | 7.00 | 6.88 | 0.35 | 6.88 | 1.00 |
| 8. Attentiveness to patients' personal experiences does not influence treatment outcomes.ᵇ | 6.88 | 7.00 | 0.35 | 6.71 | 0.17 |
| 9. Physicians should try to stand in their patients' shoes when providing care to them. | 6.25 | 6.88 | 0.35 | 6.88 | 1.00 |
| 10. Patients value a physician's understanding of their feelings which is therapeutic in its own right. | 6.38 | 6.88 | 0.08 | 6.88 | 1.00 |
| 11. Patients' illnesses can be cured only by medical or surgical treatment; therefore, physicians' emotional ties with their parents do not have a significant influence in medical or surgical treatment.ᵇ | 7.00 | 7.00 | 1.00 | 7.00 | 1.00 |
| 12. Asking patients about what is happening in their personal lives is not helpful in understanding their physical complaints.ᵇ | 7.00 | 6.63 | 0.20 | 6.86 | 0.17 |
| 13. Physicians should try to understand what is going on in their patients' minds by paying attention to their non-verbal cues and body language. | 6.50 | 5.63 | 0.33 | 6.29 | 0.38 |
| 14. I believe that emotion has no place in the treatment of medical illness.ᵇ | 6.75 | 6.88 | 0.35 | 6.88 | 1.00 |
| 15. Empathy is a therapeutic skill without which the physician's success is limited. | 6.75 | 6.13 | 0.41 | 6.71 | 0.41 |
| 16. Physicians' understanding of the emotional status of their patients, as well as that of their families is one important component of the physician-patient relationship. | 6.75 | 6.75 | 1.00 | 6.71 | 0.69 |
| 17. Physicians should try to think like their patients in order to render better care. | 5.50 | 5.50 | 1.00 | 5.86 | 0.57 |
| 18. Physicians should not allow themselves to be influenced by strong personal bonds between their patients and their family members.ᵇ | 4.50 | 4.38 | 0.85 | 3.29 | 0.28 |
| 19. I do not enjoy reading non-medical literature or the arts.ᵇ | 6.38 | 6.50 | 0.35 | 6.50 | 1.00 |
| 20. I believe that empathy is an important therapeutic factor in medical treatment. | 6.88 | 7.00 | 0.35 | 7.00 | 1.00 |
| ᵃPaired T-test |  |  |  |  |  |
| ᵇThe scores for negatively worded itemswere inverted so that higher scores indicate stronger disagreement toward these statements. |  |  |  |  |  |
| Boldface indicates statistical significance. |  |  |  |  |  |

Supplemental Table 9:

| Item Scores on the Anxiety Scale for the Pre-, Post and Delayed Post for the Disability Elective |  |  |  |  |  |
| --- | --- | --- | --- | --- | --- |
| Please for each of the items listed below, indicate how you feel when interacting with individuals with disabilities. | Pre-survey | Post-survey | Pᵃ (pre vs post) | Delayed Post-survey | Pᵃ (post vs delayed post) |
| 1. Nervous | 5.750 | 3.750 | 0.077 | 4.143 | 0.448 |
| 2. Friendlyᵇ | 3.125 | 2.250 | 0.087 | 2.000 | 0.356 |
| 3. Uncertain | 6.000 | 4.125 | 0.095 | 3.571 | 0.289 |
| 4. Comfortableᵇ | 4.500 | 4.125 | 0.697 | 3.429 | 0.200 |
| 5. Worried | 4.875 | 2.750 | **0.028** | 2.571 | 0.736 |
| 6. Trustingᵇ | 3.625 | 2.875 | 0.170 | 2.714 | 0.673 |
| 7. Threatened | 1.625 | 1.250 | 0.402 | 1.250 | 1.000 |
| 8. Confidentᵇ | 5.625 | 4.500 | 0.161 | 3.714 | 0.066 |
| 9. Awkward | 5.500 | 4.875 | 0.544 | 5.429 | 0.853 |
| 10. Safeᵇ | 3.000 | 1.875 | 0.174 | 2.143 | 0.604 |
| 11. Anxious | 5.250 | 3.500 | 0.160 | 4.286 | 0.248 |
| 12. At Easeᵇ | 5.750 | 4.250 | 0.119 | 4.250 | 1.000 |
| ᵃPaired T-test |  |  |  |  |  |
| ᵇQuestions were inverted for scoring. |  |  |  |  |  |
| Boldface indicates statistical significance. |  |  |  |  |  |

Supplemental Table 10:

| Item Scores on Comfort and Confidence Scales for the Pre-, Post and Delayed Post for the Disability Elective |  |  |  |  |  |
| --- | --- | --- | --- | --- | --- |
| Please use the scale to indicate the degree to which you agree or disagree with each statement. There are no right or wrong answers. The best response is the one that truly reflects your personal opinion. | Pre-survey | Post-survey | Pᵃ (pre vs post) | Delayed Post-survey | Pᵃ (post vs delayed post) |
| 1. I feel comfortable approaching someone with a disability who may need assistance.ᵇ | 4.00 | 4.50 | 0.17 | 4.57 | 0.36 |
| 2. I am comfortable providing assistance appropriately to a person with a disability.ᵇ | 3.00 | 4.13 | 0.05 | 4.14 | 0.69 |
| 3. I am comfortable adapting my body positions to make someone who uses a wheelchair more comfortable.ᵇ | 2.88 | 4.25 | **0.01** | 4.29 | 0.69 |
| 4. I am comfortable adapting my body positions to facilitate effective communication for someone who is visually or hearing impaired.ᵇ | 3.63 | 4.75 | **0.03** | 4.43 | 0.17 |
| 5. I am comfortable adapting my interviewing technique to accommodate patients with disabilities.ᵇ | 2.75 | 4.25 | **0.01** | 4.00 | 0.60 |
| 6. I am comfortable using appropriate communication techniques to gather accurate and complete information from patients who may be non-verbal.ᵇ | 2.75 | 3.75 | 0.19 | 3.75 | 1.00 |
| 7. I am comfortable adapting physical examination techniques to accommodate persons with disabilities.ᵇ | 2.38 | 3.50 | 0.08 | 3.86 | 0.39 |
| 8. I can see myself as an advocate for my patients with disabilities.ᵇ | 4.63 | 4.63 | 1.00 | 4.71 | 0.36 |
| 9. I feel prepared to take care of patients with disabilities.ᶜ | 2.00 | 3.63 | **0.01** | 3.63 | 1.00 |
| 10. I feel confident in communicating with patients with disabilities.ᶜ | 2.63 | 4.25 | **0.02** | 4.25 | 1.00 |
| 11. I feel confident in my understanding of "disability".ᶜ | 3.25 | 4.13 | **0.04** | 4.29 | 0.36 |
| 12. I feel confident in knowledge of barriers to access to care for persons with disabilities.ᶜ | 2.38 | 4.38 | **0.01** | 4.43 | 0.74 |
| 13. I feel confident in my understanding of the concept of ability and participation in the International Classification of Function.ᶜ | 1.50 | 4.50 | **0.00** | 4.50 | 1.00 |
| 14. I feel confident in my understanding of cultural, economic, and physical barriers to participation.ᶜ | 2.88 | 4.50 | **0.02** | 4.71 | 0.17 |
| 15. I feel confident in knowledge about my understanding about the quality of life issues for people with disability.ᶜ | 2.88 | 4.13 | **0.03** | 4.57 | 0.10 |
| ᵃPaired T-test |  |  |  |  |  |
| ᵇAdapted from a Survey Scale section ‘Interacting with the Disabled” and one question from the “Advocacy” scale (Saketkoo et al. 2004). |  |  |  |  |  |
| ᶜNewly developed confidence questions by medical education experts |  |  |  |  |  |
| Boldface indicates statistical significance. |  |  |  |  |  |

Supplemental Table 11:

| Item Scores on the Medical Student Attitudes Towards Persons with Disabilities comparing Disability Health Session vs Disability Elective Post-survey data |  |  |  |
| --- | --- | --- | --- |
| Please choose the appropriate answer choice which best corresponds with how you feel about the statement. Choose only ONE response for each question. | Post-Survey Disability Health Session | Post-survey Disability Elective | Pᵃ (disability health session vs disability elective) |
| 1. Most people with disabilities feel sorry for themselves.ᵇ | 3.296 | 3.750 | **0.036** |
| 2. I am comfortable being around a person who has an intellectual disability | 2.963 | 3.375 | 0.063 |
| 3. People with disabilities are as happy as people without disabilities. | 3.185 | 3.375 | 0.336 |
| 4. I would be comfortable interacting with a person with an intellectual disability who was in the community on his or her own (i.e, without staff members or caretakers). | 3.074 | 3.625 | **0.013** |
| 5. I would be comfortable being around a person who uses a wheelchair. | 3.537 | 3.625 | 0.684 |
| 6. Most people with disabilities resent people without disabilities.ᵇ | 3.519 | 3.750 | 0.226 |
| 7. I would be comfortable being around a person who is deaf. | 3.167 | 3.500 | 0.147 |
| 8. I am only comfortable around people with intellectual disabilities if they are well-behaved.ᵇ | 2.722 | 2.750 | 0.923 |
| 9. Most people with disabilities expect special treatment.ᵇ | 3.167 | 3.625 | 0.057 |
| 10. I would be comfortable working with a person with an intellectual disability who had someone assigned to supervise or train her. | 3.333 | 3.500 | 0.425 |
| 11. Most people with disabilities are not ashamed of their disability. | 3.111 | 3.500 | 0.061 |
| 12. If I were visited by a person who is blind, I would be comfortable helping him or her navigate the environment. | 3.333 | 3.250 | 0.726 |
| 13. I am more comfortable around people with intellectual disabilities when they have someone who is not disabled to help them.ᵇ | 2.185 | 2.000 | 0.444 |
| 15. People with disabilities should be cared for in any primary care office as opposed to a specialty clinic. | 2.830 | 3.000 | 0.566 |
| 16. I would feel comfortable living next door to a person with an intellectual disability that lives by himself. | 3.259 | 3.625 | 0.134 |
| 17. I would be comfortable living in a neighborhood where there is a group home for people with various developmental disabilities (e.g., Down Syndrome, Cerebral Palsy) | 3.481 | 3.875 | **0.050** |
| 18. I would feel comfortable being around a person with an intellectual disability in public even though his behavior might be a bit bizarre (e.g., rocking back and forth, talking loud, etc.). | 3.019 | 3.375 | 0.116 |
| Scenario A:  You enter the exam room. A middle-aged man and woman are there. He tells you he is experiencing chronic abdominal pain. |  |  |  |
| 20. In scenario A, I would be comfortable determining the role of the man vs. the woman in providing the history of the complaint. | 3.407 | 3.750 | 0.109 |
| 21. In scenario A, I would be comfortable performing a physical exam on the patient. | 3.389 | 3.625 | 0.315 |
| 22. In scenario A, I would be comfortable establishing a differential diagnosis for the abdominal pain. | 3.130 | 3.500 | 0.212 |
| Scenario B:  You enter the exam room. A middle-aged man is seated in a wheel chair. Standing behind him is a woman of about the same age. The patient in the wheel chair appears to have spasticity in all 4 limbs. He greets you by saying "hello". His speech is somewhat garbled, though intelligible. The woman tells you that the patient is here because he is experiencing chronic abdominal pain. |  |  |  |
| 24. In scenario B, I would be comfortable determining the role of the man vs. the woman in providing the history of the complaint. | 3.074 | 3.625 | **0.036** |
| 25. In scenario B, I would be comfortable performing a physical exam on the patient. | 2.407 | 2.750 | 0.224 |
| 26. In scenario B, I would be comfortable establishing a differential diagnosis for the abdominal pain. | 2.519 | 3.000 | 0.104 |
| ᵃEqual Variance T-test |  |  |  |
| ᵇThe scores for negatively worded items were inverted so that higher scores indicate stronger disagreement toward these statements. |  |  |  |

Supplemental Table 12:

| Item Scores on the Disability Attitudes In Healthcare Survey comparing Disability Health Session vs Disability Elective Post-survey data |  |  |  |
| --- | --- | --- | --- |
| Please use the scale to indicate the degree to which you agree or disagree with each statement. There are no right or wrong answers. The best response is the one that truly reflects your personal opinion. | Post-Survey Disability Health Session | Post-survey Disability Elective | Pᵃ (disability health session vs disability elective) |
| 1. People with disabilities are pleasant to be with. | 4.17 | 4.63 | 0.11 |
| 2. If I had a choice, I would rather see able-bodied patients than patients with disabilities.ᵇ | 3.31 | 3.88 | 0.15 |
| 3. It is society's responsibility to provide care for its persons with disabilities. | 4.46 | 4.38 | 0.78 |
| 4. Patients with disability improve with treatment. | 3.69 | 4.38 | **0.05** |
| 5. Medical care for PWDs uses up too many resources.ᵇ | 4.33 | 4.63 | 0.36 |
| 6. Taking a medical history from a patient with disability is frequently an ordeal.ᵇ | 3.48 | 3.88 | 0.27 |
| 7. People with disability, in general, do not contribute much to society.ᵇ | 4.52 | 4.75 | 0.45 |
| 8. People with disability don't contribute their fair share toward paying for their health care.ᵇ | 4.22 | 4.38 | 0.67 |
| 9. I will welcome patients with disability into my practice. | 4.67 | 4.75 | 0.67 |
| 10. If handled properly, the patient with disability can be seen as quickly as any other patient. | 3.72 | 2.75 | **0.03** |
| 11. Understand my patients with disability is valuable to me as a physician. | 4.74 | 4.75 | 0.96 |
| 12. Patients with disability are better off in nursing homes.ᵇ | 4.54 | 4.63 | 0.67 |
| 13. Medicare offers adequate compensation for care of patients with disability. | 2.28 | 1.25 | **0.00** |
| 14. The treatment of patients with disability is too time consuming.ᵇ | 3.94 | 4.25 | 0.33 |
| 15. More training is needed to prepare health practitioners to provide care to the patient with disability. | 4.69 | 4.88 | 0.31 |
| 16. It is unglamourous to care for people with disability.ᵇ | 3.43 | 2.38 | **0.02** |
| 17. Treatment of people with disability is hopeless.ᵇ | 4.74 | 5.00 | 0.20 |
| ᵃEqual Variance T-test |  |  |  |
| ᵇThe scores for negatively worded items were inverted so that higher scores indicate stronger disagreement toward these statements. |  |  |  |
| Boldface indicates statistical significance. |  |  |  |

Supplemental Table 13:

| Item Scores on the Jefferson Scale of Empathy for the Pre-, Post and Delayed Post for the Disability Elective |  |  |  |
| --- | --- | --- | --- |
| Please use the following 7-point scale (a higher number on the scale indicates more agreement): Mark one and only one response for each statement | Post-Survey Disability Health Session | Post-survey Disability Elective | Pᵃ (disability health session vs disability elective) |
| 1. Physicians' understanding of their patients' feelings and the feelings of their patients' families does not influence medical or surgical treatment.ᵇ | 5.981 | 6.125 | 0.734 |
| 2. Patients feel better when their physicians understand their feelings. | 6.741 | 6.875 | 0.511 |
| 3. It is difficult for physician to view things from patients' perspectives. | 4.907 | 4.375 | 0.313 |
| 4. Understanding body language is as important as verbal communication in physician-patient relationships. | 6.204 | 6.375 | 0.617 |
| 5. A physician's sense of humor contributes to a better clinical outcome. | 5.315 | 4.625 | 0.096 |
| 6. Because people are different, it is difficult to see things from patients' perspectives.ᵇ | 4.778 | 4.875 | 0.864 |
| 7. Attention to patients' emotions is not important in history taking.ᵇ | 6.259 | 6.875 | 0.225 |
| 8. Attentiveness to patients' personal experiences does not influence treatment outcomes.ᵇ | 6.333 | 7.000 | 0.031 |
| 9. Physicians should try to stand in their patients' shoes when providing care to them. | 6.074 | 6.000 | 0.827 |
| 10. Patients value a physician's understanding of their feelings which is therapeutic in its own right. | 6.167 | 6.750 | 0.113 |
| 11. Patients' illnesses can be cured only by medical or surgical treatment; therefore, physicians' emotional ties with their parents do not have a significant influence in medical or surgical treatment.ᵇ | 6.500 | 7.000 | 0.047 |
| 12. Asking patients about what is happening in their personal lives is not helpful in understanding their physical complaints.ᵇ | 6.519 | 6.625 | 0.730 |
| 13. Physicians should try to understand what is going on in their patients' minds by paying attention to their non-verbal cues and body language. | 6.056 | 5.625 | 0.426 |
| 14. I believe that emotion has no place in the treatment of medical illness.ᵇ | 6.611 | 6.875 | 0.326 |
| 15. Empathy is a therapeutic skill without which the physician's success is limited. | 6.130 | 6.125 | 0.992 |
| 16. Physicians' understanding of the emotional status of their patients, as well as that of their families is one important component of the physician-patient relationship. | 6.426 | 6.750 | 0.265 |
| 17. Physicians should try to think like their patients in order to render better care. | 5.870 | 5.500 | 0.298 |
| 18. Physicians should not allow themselves to be influenced by strong personal bonds between their patients and their family members.ᵇ | 4.574 | 4.375 | 0.763 |
| 19. I do not enjoy reading non-medical literature or the arts.ᵇ | 6.278 | 6.500 | 0.575 |
| 20. I believe that empathy is an important therapeutic factor in medical treatment. | 6.537 | 7.000 | 0.086 |
| ᵃEqual Variance T-test |  |  |  |
| ᵇThe scores for negatively worded items were inverted so that higher scores indicate stronger disagreement toward these statements. |  |  |  |
| Boldface indicates statistical significance. |  |  |  |

Supplemental Table 14:

| Item Scores on the Anxiety Scale for the Pre-, Post and Delayed Post for the Disability Elective |  |  |  |
| --- | --- | --- | --- |
| Please for each of the items listed below, indicate how you feel when interacting with individuals with disabilities. | Post-Survey Disability Health Session | Post-survey Disability Elective | Pᵃ (disability health session vs disability elective) |
| 1. Nervous | 5.185 | 3.750 | 0.053 |
| 2. Friendlyᵇ | 2.870 | 2.250 | 0.297 |
| 3. Uncertain | 6.000 | 4.125 | **0.019** |
| 4. Comfortableᵇ | 5.278 | 4.125 | **0.084** |
| 5. Worried | 4.593 | 2.750 | **0.013** |
| 6. Trustingᵇ | 3.833 | 2.875 | 0.103 |
| 7. Threatened | 2.167 | 1.250 | **0.049** |
| 8. Confidentᵇ | 5.556 | 4.500 | 0.100 |
| 9. Awkward | 5.556 | 4.875 | 0.417 |
| 10. Safeᵇ | 3.185 | 1.875 | **0.027** |
| 11. Anxious | 4.981 | 3.500 | **0.043** |
| 12. At Easeᵇ | 5.611 | 4.250 | **0.045** |
| ᵃPaired T-test |  |  |  |
| ᵇQuestions were inverted for scoring. |  |  |  |
| Boldface indicates statistical significance. |  |  |  |

Supplemental Table 15:

| Item Scores on Comfort and Confidence Scales for the Pre-, Post and Delayed Post for the Disability Elective |  |  |  |
| --- | --- | --- | --- |
| Please use the scale to indicate the degree to which you agree or disagree with each statement. There are no right or wrong answers. The best response is the one that truly reflects your personal opinion. | Post-Survey Disability Health Session | Post-survey Disability Elective | Pᵃ (disability health session vs disability elective) |
| 1. I feel comfortable approaching someone with a disability who may need assistance.ᵇ | 4.167 | 4.500 | 0.229 |
| 2. I am comfortable providing assistance appropriately to a person with a disability.ᵇ | 3.704 | 4.125 | 0.210 |
| 3. I am comfortable adapting my body positions to make someone who uses a wheelchair more comfortable.ᵇ | 3.889 | 4.250 | 0.390 |
| 4. I am comfortable adapting my body positions to facilitate effective communication for someone who is visually or hearing impaired.ᵇ | 4.111 | 4.750 | **0.045** |
| 5. I am comfortable adapting my interviewing technique to accommodate patients with disabilities.ᵇ | 3.815 | 4.250 | 0.238 |
| 6. I am comfortable using appropriate communication techniques to gather accurate and complete information from patients who may be non-verbal.ᵇ | 3.130 | 3.750 | 0.175 |
| 7. I am comfortable adapting physical examination techniques to accommodate persons with disabilities.ᵇ | 3.000 | 3.500 | 0.269 |
| 8. I can see myself as an advocate for my patients with disabilities.ᵇ | 4.000 | 4.625 | 0.070 |
| 9. I feel prepared to take care of patients with disabilities.ᶜ | 2.481 | 3.625 | **0.005** |
| 10. I feel confident in communicating with patients with disabilities.ᶜ | 3.259 | 4.250 | **0.017** |
| 11. I feel confident in my understanding of "disability".ᶜ | 3.907 | 4.125 | 0.481 |
| 12. I feel confident in knowledge of barriers to access to care for persons with disabilities.ᶜ | 3.556 | 4.375 | **0.023** |
| 13. I feel confident in my understanding of the concept of ability and participation in the International Classification of Function.ᶜ | 2.685 | 4.500 | **0.000** |
| 14. I feel confident in my understanding of cultural, economic, and physical barriers to participation.ᶜ | 3.444 | 4.500 | **0.007** |
| 15. I feel confident in knowledge about my understanding about the quality of life issues for people with disability.ᶜ | 3.407 | 4.125 | 0.063 |
| ᵃEqual Variance T-test |  |  |  |
| ᵇAdapted from a Survey Scale section ‘Interacting with the Disabled” and one question from the “Advocacy” scale (Saketkoo et al. 2004). |  |  |  |
| ᶜNewly developed confidence questions by medical education experts |  |  |  |
| Boldface indicates statistical significance. |  |  |  |
