# Supplementary material for: Disability health in medical education: development, implementation, and evaluation of a pilot curriculum at Stanford School of Medicine
Source: Front Med (Lausanne). 2024 Sep 4;11:1355473. doi: 10.3389/fmed.2024.1355473 (PMC11408233; doi:10.3389/fmed.2024.1355473)
Supplement: SUPPLEMENTARY DATA SHEET 2 — Supplemental Appendix 1. [file Data_Sheet_2.DOCX]

**Appendix A: Disability Health Session Facilitator Guide and Cases**

| **FACILITATOR NOTES** |
| --- |

**Ground rules for today:**

1. **This is a safe learning environment**
2. **Use respectful tone and language**
3. **Ask questions when you do not understand; do not assume you know what others are thinking**
4. **Maintain confidentiality**

**Students are expected to do the reading for each small group IN ADVANCE of the session. Facilitators preceptors should therefore not spend time reviewing basic concepts covered in the preparatory materials. The time should be used for discussion of the cases. In order to conduct this discussion and review, it is strongly suggested that each preceptor review the readings as well.**

**This is the second week of POM Quarter 2 (all students will have taken POM Quarter 1 in the fall). This is also the third session in the “Cultural Competency and Diversity” thread.**

**Definitions**

**Disability:**

- ADA (Americans with Disabilities Act) definition (<https://adata.org/faq/what-definition-disability-under-ada>):
  - The ADA defines a person with a disability as a person who has a physical or mental impairment that substantially limits one or more major life activity. This includes people who have a record of such an impairment, even if they do not currently have a disability. It also includes individuals who do not have a disability but are regarded as having a disability. The ADA also makes it unlawful to discriminate against a person based on that person’s association with a person with a disability.
  - Major life activities include, but are not limited to, caring for oneself, performing manual tasks, seeing, hearing, eating, sleeping, walking, standing, lifting, bending, speaking, breathing, learning, reading, concentrating, thinking, communicating, and working.
  - Major Bodily Functions include, but are not limited to, functions of the immune system, normal cell growth, digestive, bowel, bladder, neurological, brain, respiratory, circulatory, endocrine, and reproductive functions.
- Social security definition (<https://www.ssa.gov/disability/professionals/bluebook/general-info.htm>):
  - For all individuals applying for disability benefits under title II, and for adults applying under title XVI, the definition of disability is the same. The law defines disability as the inability to engage in any substantial gainful activity (SGA) by reason of any medically determinable physical or mental impairment(s) which can be expected to result in death or which has lasted or can be expected to last for a continuous period of not less than 12 months.
  - Under title XVI, a child under age 18 will be considered disabled if he or she has a medically determinable physical or mental impairment or combination of impairments that causes marked and severe functional limitations, and that can be expected to cause death or that has lasted or can be expected to last for a continuous period of not less than 12 months.
- IDEA (Individuals with Disabilities Education Act) definition (<https://www.ericdigests.org/1999-4/ideas.htm>):
  - The term "child with a disability" means a child: "with mental retardation, hearing impairments (including deafness), speech or language impairments, visual impairments (including blindness), serious emotional disturbance, orthopedic impairments, autism, traumatic brain injury, other health impairments, or specific learning disabilities; and who, by reason thereof, needs special education and related services."
- ICF definition (<https://www.cdc.gov/nchs/data/icd/icfoverview_finalforwho10sept.pdf>):
  - The International Classification of Functioning, Disability and Health (ICF) is a framework for describing and organizing information on functioning and disability. It provides a standard language and a conceptual basis for the definition and measurement of health and disability. The ICF integrates the major models of disability. It recognizes the role of environmental factors in the creation of disability, as well as the relevance of associated health conditions and their effects.
  - In the ICF, functioning and disability are multi-dimensional concepts, relating to:
    - The **body functions and structures of people**, and impairments thereof (functioning at the level of the body);
    - The **activities of people** (functioning at the level of the individual) and the activity limitations they experience;
    - The **participation** or involvement of people in all areas of life, and the participation restrictions they experience (functioning of a person as a member of society)
    - The **environmental factors** which affect these experiences (and whether these factors are facilitators or barriers)
  - The ICF conceptualizes a person's level of functioning as a dynamic interaction between her or his health conditions, environmental factors, and personal factors
  - It is a biopsychosocial model of disability, based on an integration of the social and medical models of disability.
  - As illustrated in Figure 1, disability is multidimensional and interactive. All components of disability are important and any one may interact with another. Environmental factors must be taken into consideration as they affect everything and may need to be changed.

- - Although personal factors are recognized in the interactive model shown in Figure 1, they are not classified in the ICF at this time. Such factors influence how disability is experienced by the individual and some, such as age and gender, are commonly included in data collections. The ICF can provide or
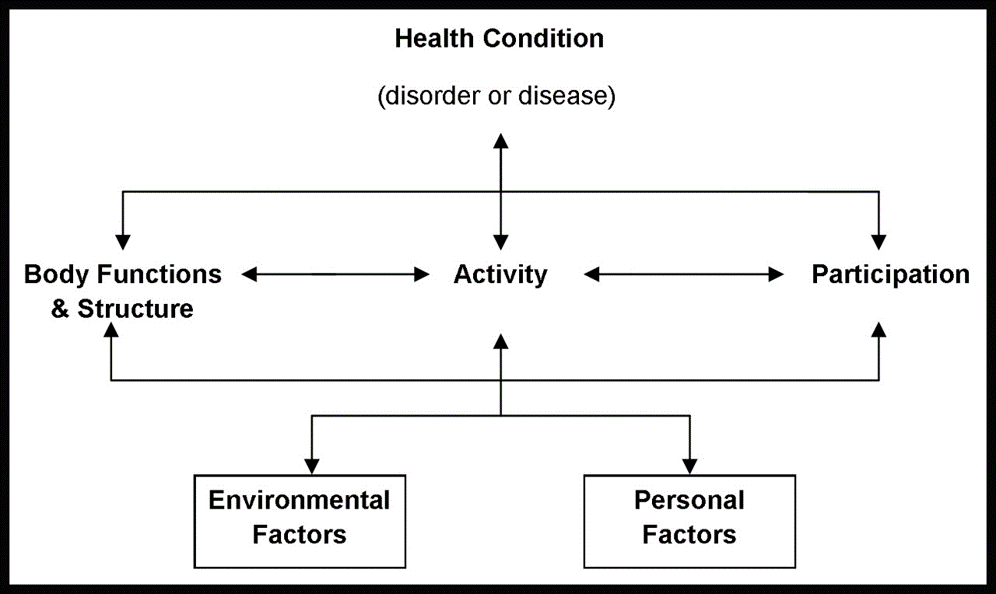
underpin a descriptive profile of an individual's pattern of functioning, not a 'yes' or 'no' answer about whether he or she is disabled. A decision about where to draw a line between ‘no disability’ and ‘disability’ depends on the purposes for doing so. Individual measures, surveys and other applications must be based on this understanding as well as the knowledge that there are multiple dimensions of disability, and potentially multiple perspectives to consider. Different measurement or policy purposes may result in different decisions about which aspects of disability to focus on and which thresholds are relevant – and hence in different measures and estimates at individual or population level

**Case 1 Scenario: John Wu**

**Case 1**, Page 1:

You are working in an outpatient clinic. The Medical Assistant (MA) tells you your next patient John Wu is ready and waiting to be seen in the clinic room. He states that the patient is 19 years old, has Down Syndrome, and is accompanied by an older woman. The chief complaint is abdominal pain. The MA describes the patient as “weeping” in pain.

The patient’s vital signs are:

Temp: 38.3 C (101F), HR: 100, RR 18, BP: 120/80, PO2 99% on RA, Height: 5’ 8’’ Weight: 170 lbs.

1. What are you thinking about when you enter the room to see John? Do you feel any discomfort or nervousness to interact with someone with Down Syndrome?
2. What assumptions could be made about John and his companion?
   - *John is likely to function in the range of intellectual disability, however, providers should not make assumptions about intelligence and competence prior to meeting a patient. Most individuals with Down syndrome function in the range of mild (IQ: 55–69) or moderate (IQ: 40–54) intellectual disability. However, a few individuals with Down syndrome score higher on IQ tests and some have severe (IQ: 20–35) intellectual disability.*
   - *Possible assumptions include: assuming that the patient wants another individual in the room both from a social/legal perspective, assuming that an adult with him is a family member. She could be a professional or a driver. Discuss why this may be problematic. Emphasize importance of obtaining a basic understanding of the relationship between the patient and anyone accompanying them.*
   - *Another assumption: Failure to address the patient directly and rather addressing the woman with him. Always address the patient directly.*
   - *Bringing an attendant is not an indication of a lower level of capacity.*
3. How will you introduce yourself to John and his companion? What if any challenges may you experience in communication with John?
   - *Offer to shake hands even if your patient has limited use of hands. Introduce yourself directly to the patient using your usual speaking voice and listen attentively for your patient to respond and to finish. If your patient appears to be thinking, wait quietly.*
   - *Address the patient like you would any other patient, even if he functions in the range of intellectual disability. Use eye contact and pay attention to nonverbal communication.*
   - *Ask the patient about his companion. Ask the person: “Do you want your support worker to stay here for this visit?” Assure that she has his permission to be in the room. Make no assumptions. This may be a professional, such as a social worker, or the person who transported him to the clinic.*
   - *Individuals with Down syndrome may have stronger receptive language than expressive language. Therefore, they may understand more than they can express to you.*
4. What are the best ways to establish rapport with a patient who has a developmental disability?
   - *Get your patient’s attention before speaking to them by using their name or by touching their arm prior to speaking*
   - *Show warmth and a positive regard*
   - *Use person‐first language (person with a disability), or identity‐first language (Autistic person), depending on patient preference. (in this case, usually person with Down Syndrome)*
   - *With patients who do not use spoken language to communicate use non‐verbal communication strategies, such as demonstrations, pictures, touch, gestures, and facial expressions.*
   - *People with social communication challenges may not be able to interpret facial expressions and gestures and therefore may benefit from concrete, literal explanations.*
   - *Check for understanding by summarizing and asking your patient to summarize in their own words.*
   - *If necessary, use short, concrete questions that require yes or no answers. If necessary, ask questions that can be answered non‐verbally. For example, “Show me how you say yes.”*


**Case 1**, Page 2:

You introduce yourself to John. He is holding his abdomen and clearly uncomfortable, but he is able to converse with you. You ask John who he came with and he says his mother. She nods to confirm. You ask John if he wants his mother to be present for the exam. He replies “Yes”.

You also ask John, “Who makes your medical decisions?” He replies “Mom”. When you ask, “What decisions do you normally make?” John looks confused. You ask his mother if she has conservatorship or guardianship over John, which she replies no. She is a big proponent of supported-decision making.

John tells you that he has a very bad “tummy ache”. When you ask him where the pain is, he points to the RLQ. You ask him to describe the pain. He simply says “bad”. When you ask him to rate his pain on a scale from 1-10, John does not respond. He looks confused when you ask whether the pain radiates. You ask him if anyone else at home is sick. He doesn’t answer.

1. After reading the information above, what was your reaction? What did you notice from communicating with John?
   - *John is able to respond to some of your questions, but there are some questions that he may not understand or be able to respond to. Therefore, it is important to explore alternate communication methods with him before jumping straight into asking his companion questions.*
   - *The physician inquired about legal guardianship or conservatorship since John is over the age of 18. A guardianship is a legal tool that allows one person or entity to make decisions for another. Because guardianship and conservatorship represent a severe limitation of civil rights, the construct of supported decision making is gaining acceptance. It means that the individual will participate to the extent possible in the decisions.* [*https://odpc.ucsf.edu/sites/odpc.ucsf.edu/files/pdf_docs/Training%20Materials%20for%20Professionals%20and%20Policy%20Makers.pd_.pdf*](https://odpc.ucsf.edu/sites/odpc.ucsf.edu/files/pdf_docs/Training%20Materials%20for%20Professionals%20and%20Policy%20Makers.pd_.pdf)

1. How would you go about getting more information about John’s case? What are techniques to get information from non-traditional communicators?
   - *Be flexible and adjust communication to John’s strengths.*
   - *Consider alternate communication strategies: eg. Instead of asking if his pain radiates, ask him to point/gesture if there is pain anywhere else in his belly.*
   - *Use visual pain scale with facial figures that change from smiling to crying.*
   - *Ask his mother about best ways to communicate with her son.*
   - *Check with the mother about whether John’s current state is his typical behavior. How does he communicate when he is not ill?*
   - *Speak normally (tone, volume, speed) unless asked to do otherwise.*
   - *Use concrete language (e.g. “Are you upset? Are you happy?” instead of “What are you feeling?”*
   - *Be patient – almost all non-traditional communication methods are extremely slow.*
   - *Don’t fill the “silence” while the other person is composing a thought with more of your own speech; allow both parties to say a reasonable amount.*
2. How would you balance getting information from John and his mother? What would be important information to get from her?
   - *There is a balance between asking the patient questions and talking with a caregiver. The initial acknowledgement of an individual with a disability is very important and goes towards treating all people respectfully. John will know best about his condition and a concerted effort needs to be made to communicate with him directly. People can tell when they are not respected.*
   - *Ask for permission from John as to whether you can ask his mother questions. Parents and caregivers who know their children very well can be utilized as a great resource to know about the patient’s condition. Ask the mother whether she has an idea of what is going on.*
   - *If his mother is not well informed about him, inquire about other adults, such as teachers, resident advisers in community living arrangements or supervisors on the job.*
   - *It is important to understand what the patient’s baseline level of functioning is. Once that is established, it is important to elucidate what is different from baseline.*

**Case 1,** Page 3:

Since John is not responding to some of your questions, you look for alternative ways of communicating and allowing John to express himself. You present John with a pictured pain scale and ask him about his pain. John points to the image that shows he has an equivalent of a 9/10 for pain. When you ask him when his pain started, he does not answer. You hold up a calendar and ask him the question again, and John points to the prior day. You ask John permission to ask his mother some questions. He nods. His mom explains that his pain started suddenly yesterday, and did not coincide with any specific event. No one else at home is ill and as far as she knows, there have been no illnesses at his school and work programs. She states he has been healthy with no medical issues in the past couple of months. She does not know about his bowel habits. His current behavior, the inability to answer verbally, is not typical of John when he is healthy.

You move on to the physical exam. John is in a lot of pain as he is holding his stomach.

1. How would you best approach doing a physical exam on John?
   - *As with any patient, describe in detail what you will be doing. Tell John slowly, clearly, with short sentences and point to the body part that you are going to check or mime that part of the physical exam on yourself.*
   - *Don’t start in the area where the pain is the most intense*
   - *Ask John how he normally likes physical exams performed. If you want more information, ask his mother if there are any ways that John prefers to have a physical examination.*
   - *Use concrete language (e.g. “put your coat on” instead of “get ready.”)*
2. How would you explain the next steps?
   - *Describe medical procedures, such as ultrasound, in simple terms. Avoid jargon.*
   - *Assure John that his mother can accompany him if he wishes.*
   - *Assure that you are working to reduce his pain as soon as possible.*
   - *Tell him what you are thinking. Tell his mother what you are thinking.*
   - *Check understanding. Ask the person, “Can you explain what I’m going to do and why?”*

Extra Resources:

Health Supervision of Children with Down Syndrome: <http://pediatrics.aappublications.org/content/128/2/393>

Health Watch Table - Down Syndrome: <http://ddprimarycare.surreyplace.ca/wp-content/uploads/2018/03/HWT_Down-Syndrome.pdf>

**Case 2 Scenario:** Maya Hernandez

**Case 2,** Page 1

You are currently working at an outpatient clinic. The MA tells you that your next patient Maya Hernandez is waiting to be seen in the clinic room. The MA states the patient is 28 years old and seated in a wheelchair. Her chief complaint is abdominal pain. The MA also mentions that she appears to have spasticity in all 4 limbs and her speech is somewhat difficult to understand.

The patient’s vital signs are:

Temp: 36.7C (98.1F), HR: 70, RR 18, BP 100/60. PO2 99% on RA.

1. What are you thinking about when you enter the room? Do you feel any discomfort or nervousness to interact with someone with a physical disability?
   - *Focus on the patient’s chief complaint as your initial line of questioning - that is the reason they are in the clinic.* ***Common pitfalls:*** *to focus on the patient’s disability rather than their acute condition. Often, people conflate disability with disease, and will not treat the patient’s acute condition as aggressively because they assume it is part of a chronic problem.*
   - *However, do not ignore the disability because it may factor into your plan for how to provide them the best care. The provider should find out about her disability. They can ask general questions, such as “Can you tell me more about any other health conditions you have (PMH)?” You can ask specifically about the disability. Potential phrases may include: “Tell me more about your mobility needs,” “I noticed you are using a wheelchair, can you tell me more about that?”*
2. What assumptions could be made about Maya?
   - *Her need to use a wheelchair to achieve mobility provides no additional information about her level of intellectual, language, or social skills. She may be a person who acquired a spinal cord injury as an adult with no accompanying problems in other domains. She may be a person with both a physical and intellectual disability, as can be the case for some individuals with cerebral palsy. Individuals with physical disabilities do not always have an intellectual disability.*
   - *If someone has speech that is more difficult to understand, that does not mean that the individual has an intellectual disability.*
   - *Do not assume that a person with a disability is not capable of having a physician appointment by themselves and requires another individual to help them make their medical decisions.*
3. What if any challenges may you experience in communication with Maya? How would you start the conversation?
   - I*t might take longer to communicate with Maya given her dysarthria.*
   - *Introduce yourself to the patient. Shake the patient’s hand. Address the patient like you would any other patient. Use eye contact and pay attention to nonverbal communication. Use her approach to your questions to understand more about her.*
   - *Take turns speaking – say a short amount and then give the other person a chance to speak; be careful of interrupting; try not to talk about more than one idea at a time*
   - *Do not pretend you understand what the person said if you do not. Let the person know if you don’t understand something.*
   - *Acknowledge and rephrase communication attempts. When responding to a question, include the question in your response to let the person know that you heard and understood them.*
4. What is important etiquette for individuals who use assistive equipment?
   - *Treat assistive devices, such as wheelchairs, as personal space. Don’t touch a wheelchair without permission.*
   - *Before helping, offer assistance and wait for a response and instructions*
   - *Do not remove the assistive equipment from the person.*
5. What’s missing in the vitals signs from MA’s report?
   - *Notice there is no weight. It is important to take complete vital signs even if it may be more difficult. The clinic may not have a mechanism for weighing individuals who use wheelchairs. Such a situation is very poor medical practice. Weight is important for health maintenance and wellness and also for monitoring in many different conditions. You need the right equipment and training for the MA.*
   - *Individuals may know how much their wheelchair weights and you can subtract if they cannot transfer out of the chair.*

**Case 2,** Page 2:

When you walk into the room you notice that the patient has difficulty controlling her muscle movements. You introduce yourself. She shakes your hand and introduces herself. She has mild dysarthria but language that can be readily understood. She confirms that her chief complaint is abdominal pain. She is alone in the room and perfectly able to provide her own history.

**HPI:** The abdominal pain started 6 weeks ago. She saw a physician about it in the clinic 3 weeks ago. That doctor stated it was likely a stomach virus, which was highly prevalent in the community. He asked her to follow-up if the pain didn’t go away. The pain has persisted to the present. The pain is diffuse and non-radiating. Patient complains of cramping and bloating, as well as some nausea and vomiting. The patient has not eaten anything differently than usual and has not traveled out of the country. No one with whom she lives is ill. She has had no change in bowel or bladder habits.

**PMH:** When you ask her why she uses the wheelchair, she states that she has cerebral palsy. She uses a wheelchair for mobility in the community and walks with canes at home. She has been very healthy. She doesn't think her abdominal pain has anything to do with her disability and describes it as cramping pain, like her monthly menstrual periods. She is on no medications.

**SH:** She is a full time master’s degree candidate.

1. Were you surprised by any part of the patient’s history?
   - *The patient was able to provide a thorough history.*
   - *The patient is pursuing a full time master’s degree.*
   - *The patient recently saw a physician about the same chief complaint.*
2. What additional information would be pertinent in this case?
   - *Abdominal pain generates a broad differential diagnosis.*
   - *In this case, we have no information about the woman’s sexual history. Assumptions are often made that individuals with disabilities do not have healthy sexual relationships*
   - *Women with disabilities receive less screening for pap smears, mammograms and decreased preventive care.*
   - *Questions should include:*
     - *LMP (last menstrual period)*
     - *Sexual history*
3. What are the top 4 items on your differential diagnosis?
   - *Pregnancy including ectopic pregnancy, sexually transmitted infection, lower tract UTI, appendicitis, diverticulitis, ovarian cyst, endometriosis.*

**Case 2**; Page 3: You take a sexual history and find out that Maya has been having sexual intercourse with a single partner, her boyfriend Steve, for the past 2 years. They have been relying on condoms for birth control. Her last menstrual period was 10 weeks ago. She has never been pregnant.

You ask her what it would mean for her if she found out she was pregnant. She said that she would be delighted. She and Steve have plans to be married next year. They are in a committed relationship. They have discussed how they both want a big family. The timing would be good because she will finish her program in 2 months and could even start a job prior to the birth.

You state that next you will be performing a physical exam. You ask how she normally moves from her chair to the exam table. She states that she can get onto the exam table on her own.

PE: She is well appearing. There are no apparent physical exam findings. Her abdomen is nontender. You discuss with her the importance of getting an accurate weight. She tells you how she can transfer from the wheelchair to the scale.

Questions:

1. Why is it important to have the patient be examined on the exam table and not in their wheelchair? How as a physician are you expected to accommodate?
   - *Examining a patient in their wheelchair is less thorough than on the exam table, and does not provide the patient equal medical services. It is hard to examine the abdomen in a seated position. There are some power wheelchairs which have the ability to recline, so if the patient has one of those then it may be possible for them to remain in their chair. There are several ways to make the exam table accessible to a person using a wheelchair. A good option is to have a table that adjusts down to the level of a wheelchair, approximately 17-19 inches from the floor. What is important is that a person with a disability receives equal medical services to those received by a person without a disability. If the examination does not require that a person lie down (for example, an examination of the face), then the exam table is not important to the medical care and the patient may remain seated. It is most important to ask the patient what they prefer in terms of performing the exam.* (<https://www.ada.gov/medcare_mobility_ta/medcare_ta.htm>)
   - *The provider must provide reasonable assistance to enable the individual to receive medical care. This assistance may include helping the patient to undress and dress, get on and off the exam table or other equipment, and lie back and be positioned on the examination table or other equipment. Once on the exam table, some patients may need a staff person to stay with them to help maintain balance and positioning. The provider should ask the patient if he or she needs any assistance and, if so, what is the best way to help. The provider should never walk away and leave the patient on the exam table.*
2. What may be issues for this woman if she is pregnant?
   - *Discussion about accessible exercise during pregnancy, and overall health and wellness*
   - *Make a plan about if she will need assistance at any point before or after the pregnancy*

Extra Resources:

Gross Motor Function Classification System (GMFCS) - <https://cpqcc.org/sites/default/files/documents/HRIF_QCI_Docs/GMFCS-ER.pdf>

Disabled and Fighting for a Sex Life - <https://www.theatlantic.com/health/archive/2015/03/sex-and-disability/386866/>

**Case 3 Scenario:**

**Case 3**: Page 1

You are currently working at an outpatient clinic. The MA tells you your next patient Jacob Thomas is waiting to be seen in the clinic room. The MA states that the chief complaint is abdominal pain. Jacob is 18 years old. His father is in the waiting room, as Jacob requested. The father told the MA that his son has autism. The patient’s vital signs were:

Temp: 36.7C (98.1F), HR: 80, RR 18, BP 115/72, PO2 99% on RA, Ht 6 foot, Wt 65 kg.

1. What are you thinking about when you enter the room to see Jacob?
2. What if any challenges may you experience in communication with Jacob?
3. How will you interact with Jacob’s father?

Learning points:

- *Autism is a spectrum. You do not know from the diagnosis the level of function of the patient.*
- *Communication is likely a challenge for patients with autism spectrum disorder. Approximately 25-40% of people with autism have minimal or no verbal language. Some individuals with autism use alternative communication such as typing or a speech generation device.*
- *Social interactions are another core deficit in autism. The patient may have difficulty in relating to you.*
- *Many individuals on the autism spectrum function in the range of intellectual disability. However, some score in the average or superior range of intellectual ability.*
- *Given Jacob’s age, you will need his explicit permission to interact with his father unless his father has guardianship or legal rights of some kind.*

**Case 3,** Page 2:

When you walk in the room, you see a teenage boy sitting on the exam table, holding his stomach and moaning. As you interview Jacob about his pain, you observe he doesn’t make eye contact with you. He has several repetitive hand movements. However, he is able to answer your questions.

Jacob says that he gets this abdominal pain quite frequently, but today it is worse than usual. It gets worse during school week and is much better during weekend. Last summer the pain resolved completely but it returned this fall when school resumed.

PMH: He says he has been healthy. He is on no medications.

SH: He lives at home with his parents. His father drove him to the clinic. Since he turned 18 years old, he has not wanted his parents in the exam room with him. He is a senior in the local high school. He is in all general education classes. He is planning attend the local community college next year. However, his grades have been dropping this year. He is not sure he will be able to attend.

Questions:

1. What further information would you want to know to help understand Jacob’s situation?
2. What challenges do individuals with disabilities face in a school setting?
3. What is your differential diagnosis?
4. How would you go about getting more information?

Learning points:

- *You may need to know about his presentation before this pain. Has he had similar issues in the past? How does he respond to pain? To stress?*
- *What is going on at school? How do the other students relate to him? How happy is he at school? Considering his case from the social model of disability: how do we ensure that his environment (eg school) is accepting and accommodating of his differences?*
- *Mental health:. Incidence of mental health disorders in patients with autism is significant. Anxiety is common (roughly 40% have coexisting anxiety disorder)*
  1. *Difficulty in diagnosis*
     1. *Provider assumptions that behavioral problems are due to autism*
     2. *Communication challenges--patient may not be able to share what they are feeling. Those who can communicate well may still have difficulty identifying and understanding the emotions they are feeling (alexithymia)*
- *Consider asking his permission to talk with his father and maybe school personnel.*

Extra Resources:

- Health Watch Table - Autism Spectrum Disorder <http://ddprimarycare.surreyplace.ca/wp-content/uploads/2018/03/HWT_ASD.pdf>

**Information About the Disabilities Discussed in Cases**

**Case 1: Down Syndrome: (Health Supervision of Children with Down Syndrome -** [**http://pediatrics.aappublications.org/content/128/2/393**](http://pediatrics.aappublications.org/content/128/2/393) **)**

Children with Down syndrome have multiple malformations, medical conditions, and cognitive impairment because of the presence of extra genetic material from chromosome 21.1,2 Although the phenotype is variable, there typically are multiple features that enable the experienced clinician to suspect the diagnosis. Among the more common physical findings are hypotonia, small brachycephalic head, epicanthal folds, flat nasal bridge, upward-slanting palpebral fissures, Brushfield spots, small mouth, small ears, excessive skin at the nape of the neck, single transverse palmar crease, and short fifth finger with clinodactyly and wide spacing, often with a deep plantar groove between the first and second toes. The degree of cognitive impairment is variable and may be mild (IQ of 50 –70), moderate (IQ of 35–50), or occasionally severe (IQ of 20 –35). There is a significant risk of hearing loss (75%); obstructive sleep apnea (50%–79%); otitis media (50%–70%); eye disease (60%), including cataracts (15%) and severe refractive errors (50%); congenital heart defects (50%); neurologic dysfunction (1%–

13%); gastrointestinal atresias (12%); hip dislocation (6%); thyroid disease (4%–18%)3–6; and, less commonly, transient myeloproliferative disorder (4%–10%) and later leukemia (1%) and Hirschsprung disease (1%). The social quotient may be improved with early-intervention techniques, although the level of function is exceedingly variable. Children with Down syndrome often function more effectively

in social situations than would be predicted on the basis of cognitive assessment results.

Medical management, home environment, early intervention, education, and vocational training can significantly affect the level of functioning of children and adolescents with Down syndrome and facilitate their transition to adulthood. The following outline is designed to help the pediatrician provide care for children with Down syndrome and their families in the medical home. It is organized by the issues that need to be addressed in various age groups (see Appendix 1). Several areas require ongoing assessment throughout childhood and should be reviewed at every physician visit and at least annually. These areas include:

● personal support available to family;

● participation in a family-centered medical home;

● age-specific Down syndrome–related medical and developmental conditions;

● financial and medical support programs for which the child and family may be eligible;

● injury and abuse prevention with special consideration of developmental skills; and

● nutrition and activity to maintain appropriate weight.

**Case 2: Cerebral Palsy: (Cerebral palsy: Clinical features and classification - Uptodate)**

Cerebral palsy (CP) refers to a heterogeneous group of conditions involving permanent nonprogressive central motor dysfunction that affect muscle tone, posture, and movement. These conditions are due to abnormalities of the developing fetal or infantile brain resulting from a variety of causes. The motor impairment generally results in limitations in functional abilities and activity which can range in severity. Multiple additional symptoms often accompany the primary motor abnormalities, including altered sensation or perception, intellectual disability, communication and behavioral difficulties, seizure disorders, and musculoskeletal complications. Although the underlying etiology itself is not progressive, the clinical expression may change over time as the nervous system matures.

CP is characterized by abnormalities of motor activity and posture. In affected patients, voluntary movements that should be complex, coordinated, and varied are instead uncoordinated, stereotypic, and limited. Simple actions that are performed unconsciously by unaffected individuals require marked effort and concentration and often fail in patients with CP. In severely affected individuals, an attempted voluntary movement may evoke a primitive reflex, co-contraction of agonist and antagonist muscles, and mass movements [[3](https://www-uptodate-com.laneproxy.stanford.edu/contents/cerebral-palsy-clinical-features-and-classification/abstract/3)]. For example, attempts at flexion of one particular joint may involve all segments of a limb, and extension of all the fingers may accompany extension of the wrist. Discrete movements, such as that of an individual finger, may be impossible.

**Case 3: Autism (National Institute of Mental Health (NIMH)**

Autism spectrum disorder (ASD) is a term for a group of developmental disorders described by:

• Lasting problems with social communication and social interaction in different settings

• Repetitive behaviors and/or not wanting any change in daily routines

• Symptoms that begin in early childhood, usually in the first 2 years of life

• Symptoms that cause the person to need help in his or her daily life

The term “spectrum” refers to the wide range of symptoms, strengths, and levels of impairment that

people with ASD can have. The diagnosis of ASD now includes these other conditions:

• Autistic disorder

• Asperger’s syndrome

• Pervasive developmental disorder not otherwise specified

Although ASD begins in early development, it can last throughout a person’s lifetime.

What are the signs and symptoms of ASD? Not all people with ASD will show all of these behaviors, but most will show several. People with ASD may:

• Repeat certain behaviors or have unusual behaviors

• Have overly focused interests, such as with

moving objects or parts of objects

• Have a lasting, intense interest in certain topics,

such as numbers, details, or facts

• Be upset by a slight change in a routine or being

placed in a new or overstimulating setting

• Make little or inconsistent eye contact

• Tend to look and listen less to people in their environment

• Rarely seek to share their enjoyment of objects or activities by pointing or showing things to others

• Respond unusually when others show anger, distress, or affection

• Fail or be slow to respond to their name or other verbal attempts to gain their attention

• Have difficulties with the back and forth of conversations

• Often talk at length about a favorite subject but won’t allow anyone else a chance to respond or notice when others react indifferently

• Repeat words or phrases that they hear, a behavior called echolalia

• Use words that seem odd, out of place, or have a special meaning known only to those familiar with that person’s way of communicating

• Have facial expressions, movements, and gestures that do not match what they are saying

• Have an unusual tone of voice that may sound sing-song or flat and robot-like

• Have trouble understanding another person’s point of view, leaving him or her unable to predict or understand other people’s actions

People with ASD may have other difficulties, such as sensory sensitivity (being sensitive to light, noise, textures of clothing, or temperature), sleep problems, digestion problems, and irritability. People with ASD can also have many strengths and abilities. For instance, people with ASD may:

• Have above-average intelligence

• Be able to learn things in detail and remember information for long

periods of time

• Be strong visual and auditory learners

• Excel in math, science, music, and art

**Communication Tips:**

(**Tools for the primary care of adults with intellectual and developmental disabilities -** [**http://ddprimarycare.surreyplace.ca/tools-2/**](http://ddprimarycare.surreyplace.ca/tools-2/) **)**


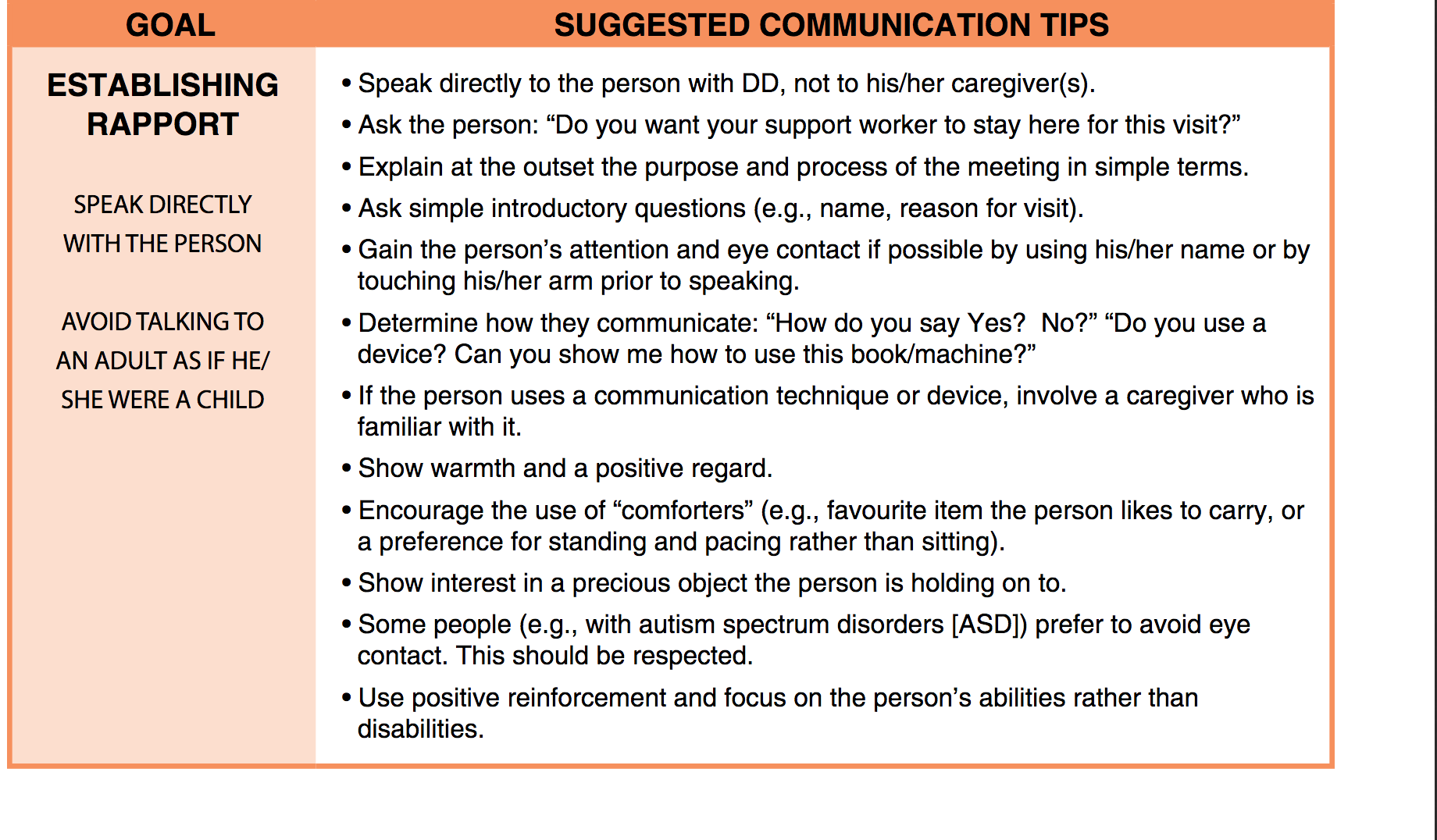


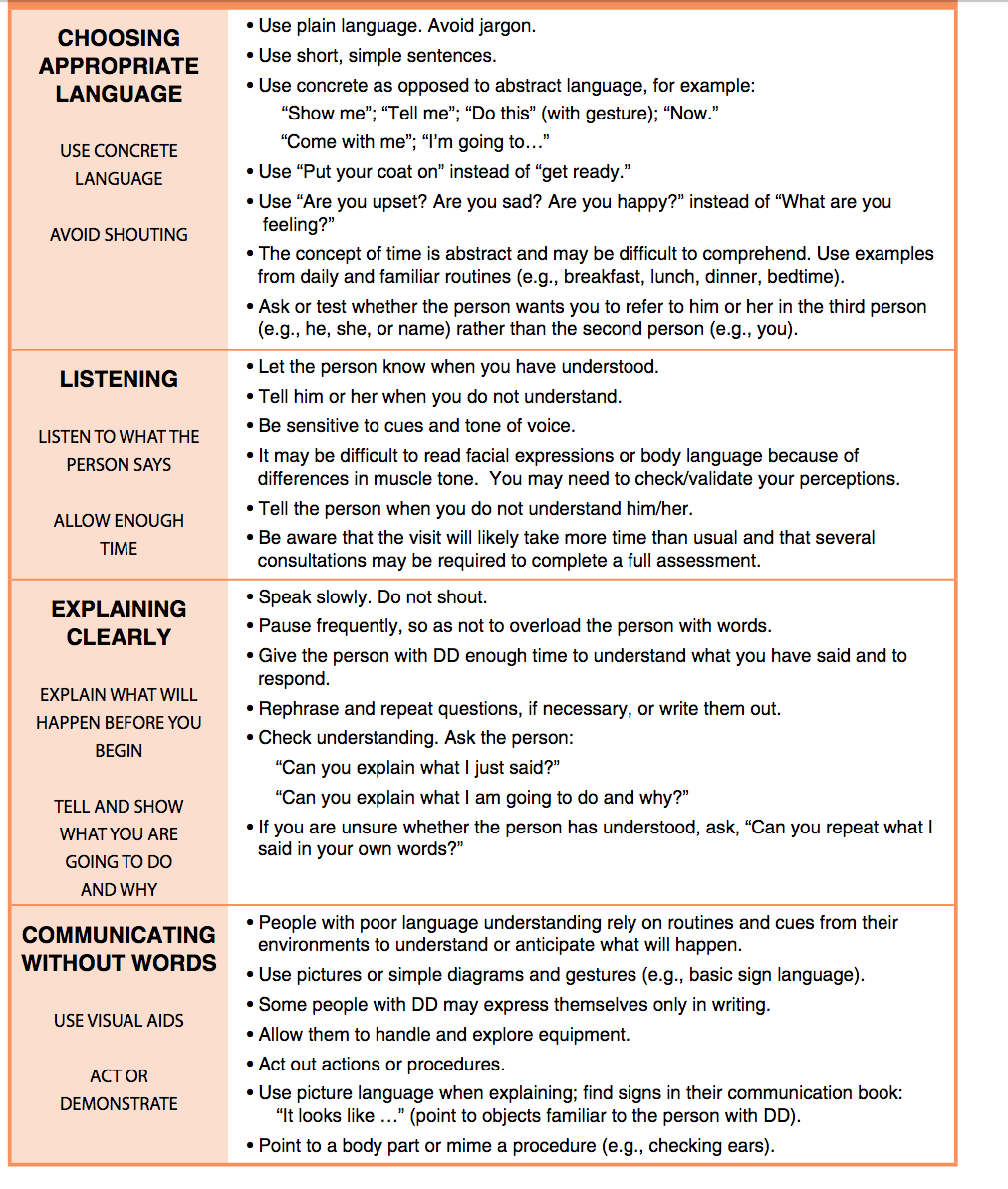


**
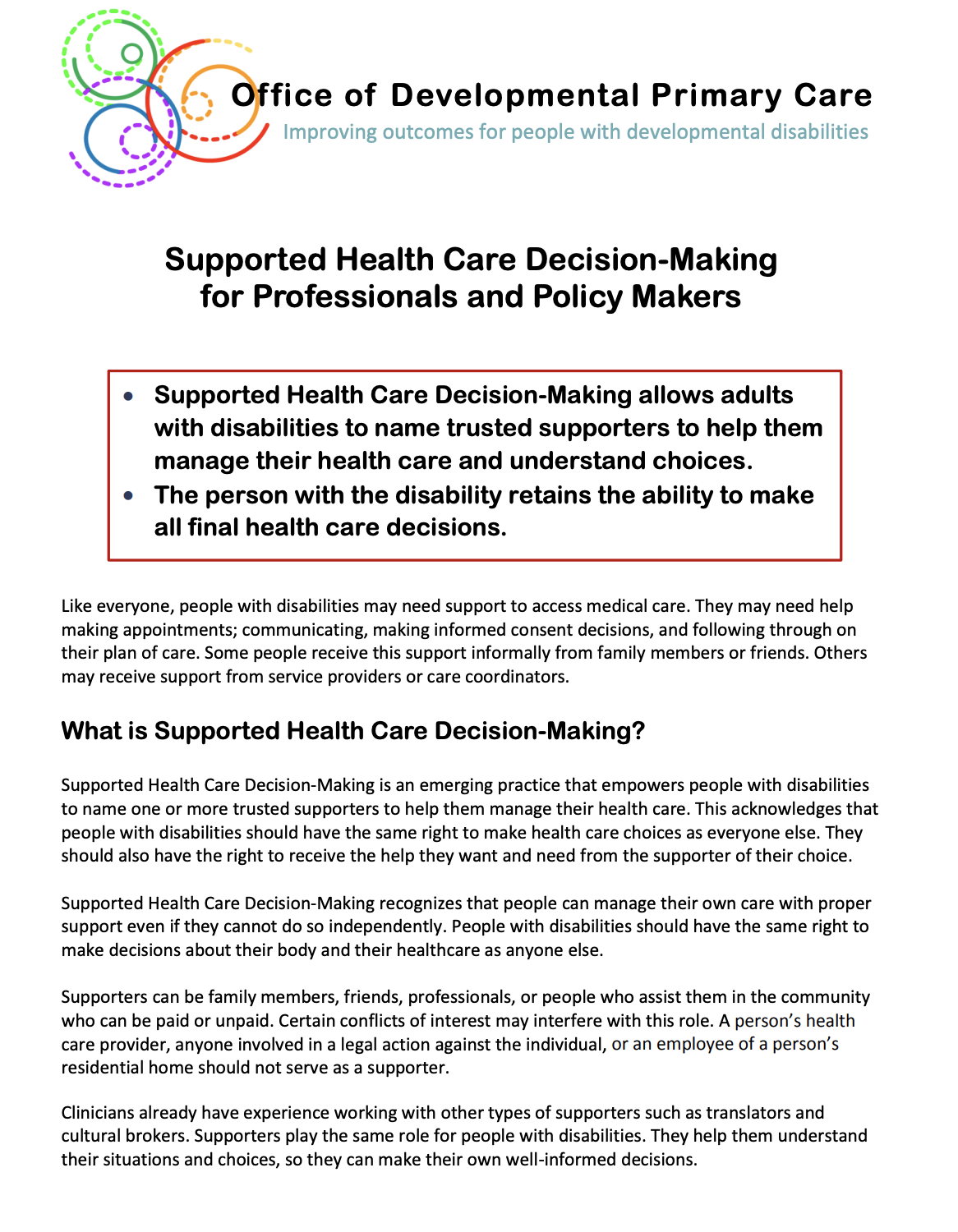

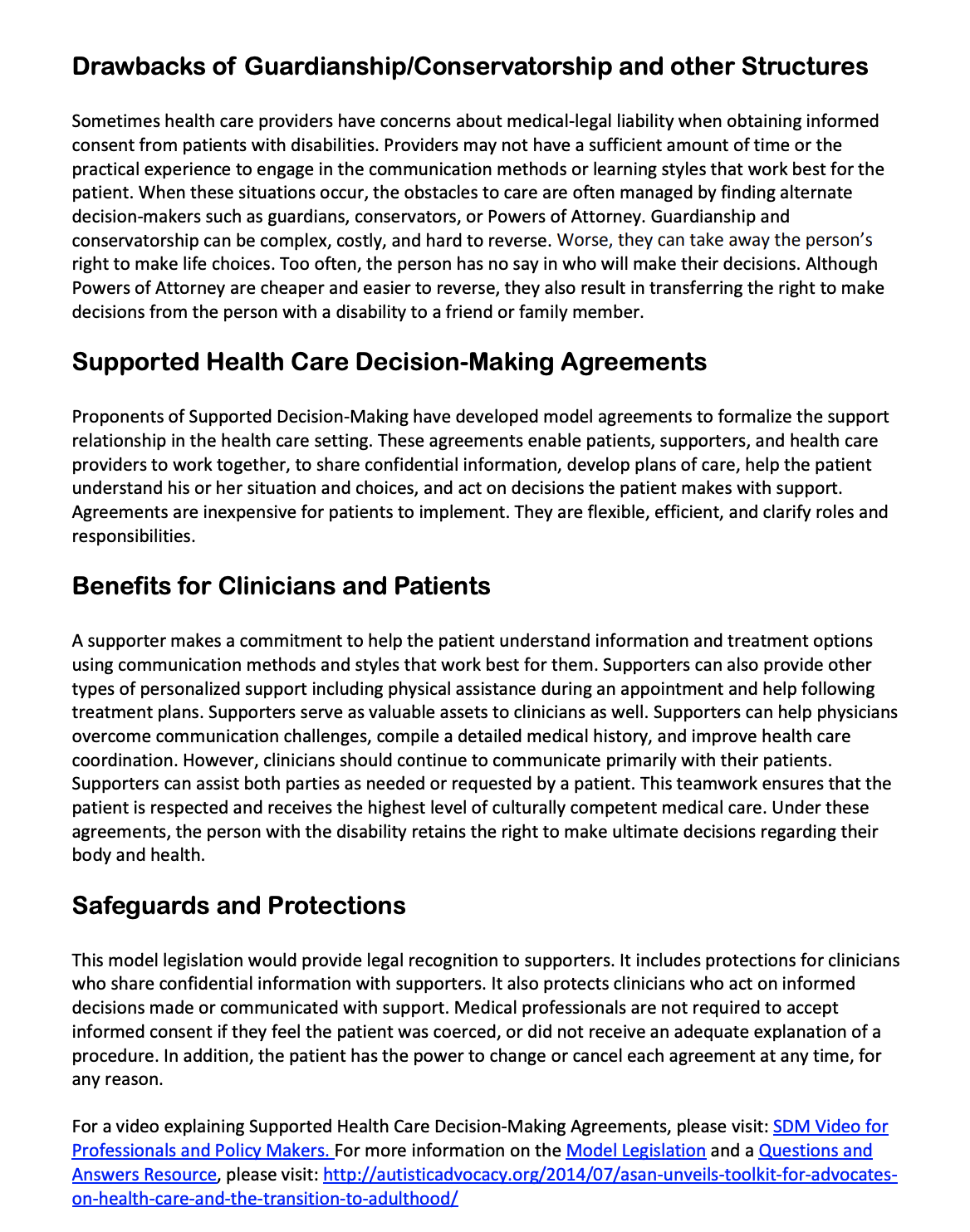
**
